# Chemical Kinetics Roots and Methods to Obtain the Probability Distribution Function Evolution of Reactants and Products in Chemical Networks Governed by a Master Equation

**DOI:** 10.3390/e21020181

**Published:** 2019-02-14

**Authors:** José-Luis Muñoz-Cobo, Cesar Berna

**Affiliations:** 1 Department of Chemical and Nuclear Engineering, Universitat Politècnica de València, 46022 Valencia, Spain; 2 Instituto Universitario de Ingeniería Energética, Universitat Politècnica de València, 46022 Valencia, Spain

**Keywords:** maximum entropy principle, chemical master equation, chemical propensity, updating probability distribution functions, chemical reaction networks

## Abstract

In this paper first, we review the physical root bases of chemical reaction networks as a Markov process in multidimensional vector space. Then we study the chemical reactions from a microscopic point of view, to obtain the expression for the propensities for the different reactions that can happen in the network. These chemical propensities, at a given time, depend on the system state at that time, and do not depend on the state at an earlier time indicating that we are dealing with Markov processes. Then the Chemical Master Equation (CME) is deduced for an arbitrary chemical network from a probability balance and it is expressed in terms of the reaction propensities. This CME governs the dynamics of the chemical system. Due to the difficulty to solve this equation two methods are studied, the first one is the probability generating function method or z-transform, which permits to obtain the evolution of the factorial moment of the system with time in an easiest way or after some manipulation the evolution of the polynomial moments. The second method studied is the expansion of the CME in terms of an order parameter (system volume). In this case we study first the expansion of the CME using the propensities obtained previously and splitting the molecular concentration into a deterministic part and a random part. An expression in terms of multinomial coefficients is obtained for the evolution of the probability of the random part. Then we study how to reconstruct the probability distribution from the moments using the maximum entropy principle. Finally, the previous methods are applied to simple chemical networks and the consistency of these methods is studied.

## 1. Introduction

The early studies on stochastic kinetics and the chemical master equation (CME) started in the early sixties with the works of McQuarrie and Montroll [1,2], followed two year later by the paper of Nicolis and Babloyantz [3]. Then, Nicolis and Prigogine [4], Nicolis and Malek-Mansour [5], Gillespie [6,7] and Van Kampen [8] obtained the complete form of the CME and, at the same time, these researchers developed tools for solving this equation at different degrees of approximation, for several simple chemical network cases. 

The chemical reaction networks can be considered as Birth-and-Death processes without memory, which are Markov processes in a multidimensional space, this point of view was studied by Anderson [9] and Van Kampen [8]. While the roots to formulate properly the CME based on microphysical principles were established by Gillespie [6,7]. 

Concerning the solution methods of the CME, due to the complexity of this equation even for well stirred chemical reacting systems, the first attempts relied upon the use of Monte-Carlo methods to sample on the underlying probability distribution, and this kind of method has been used by many authors such as Salis and Kaznessis [10], Cao, Gillespie and Petzold [11]. Gillespie developed the stochastic simulation algorithm (SSA [7]), which uses the Monte-Carlo method as a procedure for numerically simulating well-stirred chemically reacting systems by stepping in time to successive molecular reaction events in exact accord with the premises of the CME. An alternative method, which does not require generating a large number of Monte-Carlo simulations, is the Finite State Projection method (FSP) developed originally by Munsky and Khammash; this algorithm provides a direct solution to the CME, obtaining the probability density vector at a given time directly at a desired order of accuracy [12]. Other authors such as Smadbeck, Sotiropuos and Kaznessis, [13,14] prefer to obtain from the CME, the equations for the moment evolution and solve the moment equations, reconstructing at posteriori the probability density from the moments using the maximum entropy principle (MEP). Proceeding in this way, a set of coupled differential equations for the moments is obtained, but the inconvenience is that higher order moments appear. The presence of these higher moments in the differential equation system leads to the necessity of higher moment’s closure. If the reaction network system is non-linear, then in the moment equations of a given order terms with higher order moments appear, and the size of the differential equation system for the complete hierarchy of moments involved becomes infinite. The effect produced by these higher moment terms can be approximated as performed by Sotiropoulos and Kaznessis [14], and Gillespie [15], expressing these higher moments in terms of lower ones, which is known as the closure problem. In addition, Grima has studied the closure problem using the cumulant closure technique that assumes that all the cumulants of the probability distribution function (PDF), which are higher than a certain order, are negligibly small [16]. Grima in his paper has studied the accuracy of the two-moment approximation (third and higher order cumulants are zero) and of the three-moment approximation (fourth and higher order cumulants are zero) for some specific chemical systems. Also, Grima has shown [16] that for a class of chemical systems, the linear noise approximation (LNA) gives results for the mean concentrations and second moments of fluctuations about the means, which match those given by the CME for all volumes, even though the chemical system is composed of at least one second order reaction. It is convenient to remind that the LNA originates from the system-size expansion first introduced by Van Kampen [8], being the LNA the leading-order term in this expansion. The LNA transforms the chemical master equation into a Fokker-Plank equation [17]. However, this last equation gives only the time evolution of the noise PDF about the macroscopic concentrations predicted by the rate equations (REs). The inconvenience of this approach is that does not provide any correction to the macroscopic concentrations obtained from the REs and gives only an estimation of the fluctuations about the macroscopic concentrations. However, as found also by Grima [18] for some chemical systems as some simple biological reactions catalyzed by enzymes inside intracellular compartments, the time evolution of the true concentrations is not governed by the conventional rate equations but rather by a more general kind of rate equations known as the effective mesoscopic rate equations (EMREs). To deduce these effective rate equations Grima [18] in is seminal paper performs first a Van Kampen system size expansion expressing in the CME the number of molecules of each species *i* as: $\frac{N_{i}}{\Omega }=\phi_{i}+\Omega^{-1/2}\eta_{i}$, being $\phi_{i}$ the macroscopic concentration of the species *i*-th as determined by the rate equations, $\eta_{i}$ a random variable that gives the fluctuations about the value obtained from the REs, and finally Ω is the system volume. Then he expands the microscopic rate functions in the CME retaining up to second order terms in the fluctuations. In addition, he simplifies the resulting equation for the PDF of the fluctuations on account of the classical macroscopic rate equations. Finally, he uses the time evolution equation for the fluctuation PDF, to derive a set of coupled differential equations for the first and second moments of these fluctuations. Finally by averaging $\frac{N_{i}}{\Omega }$ it is obtained that the true concentration $\langle \frac{N_{i}}{\Omega }\rangle =\phi_{i}+\Omega^{-1/2}\langle \eta_{i}\rangle$, is related to the first moment of the fluctuations $\langle \eta_{i}\rangle$ and to the concentration $\phi_{i}$ as determined from the rate equations, so solving for the first moment of the fluctuation it is obtained a set of evolution equations for the true moment $\langle \frac{N_{i}}{\Omega }\rangle$, which are known as the effective mesoscopic rate equations (EMREs).

In addition, Schnoerr, Sanguinetti, and Grima performed a comparison of different moment-closure approximations for stochastic chemical kinetics [19]. Also, these same three authors performed an extensive tutorial review on the approximations and inference methods used for stochastic biochemical kinetics including among others the chemical Langevin equation, the Fokker-Plank equation, the system size expansion, and the different classes of existing moment closure approximations as normal, Poisson, Log-Normal, and derivative matching [20]. Other methods as hybrid methods have been developed by Hasenauer et al. [21,22]. Finally, we cannot forget the set of works performed by Hespanha and Singh [23,24], on the moment closure technique based on derivative-matching, which closes the moment equations by approximating higher-order moments as nonlinear functions of lower-order ones. Also, it is very original the approach of Ruess et al. [25] that perform the closure of the moment equations by replacing the moments of order higher than the closure order n by estimates calculated from a small number of stochastic simulations runs. The resulting stochastic system is then used in an extended Kalman filter, where estimates of the moments of order up to n, obtained from the same simulation, serve as outputs of the system.

Concerning the solution of stochastic chemical kinetics using different approximations to the CME the CERENA toolbox (Chemical Reaction Toolbox Analyzer) developed at the Institute of Computational Biology in Munich deserves to be mentioned [26]. This toolbox incorporates in addition to the classical stochastic simulation algorithms as the SSA, the moment method (MM) at various approximation orders, and the novel conditional moment equations for a hybrid description. This last method combines a microscopic description of low copy-number species with the classical moment description for high copy-number species, providing in this way a hybrid approach to obtain an approximate solution of the CME.

The application of the MEP provides a method to obtain the PDF of the unknown distribution variables when partial information such as some distribution moments and their support are known. The methodology consists of looking for the PDF that maximizes the Shannon information entropy [27] and, at the same time, fulfills the set of restrictions imposed by the known moments. These PDFs reconstruction methods were initiated by Shannon and Jaynes [27,28,29,30] and then were further elaborated by Mead and Papanicolau [31], Montroll and Shlesinger [32], Shore and Johnson [33], Muñoz-Cobo et al. [34], among other researchers. 

The goal of this paper is to present a unified perspective of the chemical reaction networks as birth and death Markov processes in a multidimensional vector space. Beginning with the definition of the propensities at a microscopic level in a well stirred tank, which permits the expression of the propensities in terms of microscopic and macroscopic parameters as explained in detail in Section 2 for bimolecular reactions. Another goal of this paper is to study, in a general way, the main methods to obtain approximate solutions of the CME and to make a study of the consistency of these methods, by performing cross comparisons among them. This paper has been organized as follows: Section 2.1 is devoted to presenting some introductory concepts on chemical reaction networks in multidimensional space, like the degree of advance of the chemical reactions, the rate of entropy production, the reaction affinities, and some non-equilibrium thermodynamics concepts. In Section 2.2, we study the probability distribution of the number of molecules of each chemical compound at equilibrium conditions. In Section 2.3 we study the roots of chemical kinetics from a microscopic point of view and we finish this section with the calculation of the propensities for arbitrary chemical reactions with known stoichiometric coefficients in well stirred tanks. Finally, Section 2.4 is devoted to the formulation of the CME of a reaction network and Section 2.5 to the limitations of this formulation.

Section 3 is devoted to the methods to obtain the moments of the probability distribution in chemical reaction networks, two methods are studied in this section. The first one, which is described in detail in Section 3.1, is the method of the probability generating function or z-transform. It is obtained in this section the equation obeyed by the probability generating function for the reactant and product population of the network, while Section 3.2 is devoted to show the method of the expansion of the master equation in one order parameter, general equations are obtained at different orders of approximation in terms of multinomial coefficients.

In Section 4, we study the methods to reconstruct the probability distribution function (PDF) from the moments using the maximum entropy principle. Finally, Section 5 is devoted to the application of the previous methods to study several simple reaction networks with non-linear effects in open and closed systems. Also, in this section we check the consistency of some of the approximations performed, finishing this section with the closure problem and discussing how to perform the closure in an optimal way.

## 2. Introductory Concepts on Chemical Reactions Networks and the Chemical Master Equation

In this section, we give a brief introduction about the main concepts on chemical reaction kinetics, chemical reaction networks, chemical reaction propensities, to finish with the chemical master equation and its limitations.

### 2.1. Some Introductory Concepts about Chemical Reactions Networks and Entropy Production

Let us consider a stirred tank of given volume Ω containing $n_{S}$ chemical compounds or species $X_{j}$. Let us denote by $N_{j}$ the number of molecules of the *j*-th chemical compound. The number of molecules of all the chemical species will be represented as a point N belonging to a ${\mathbb{N}}^{n_{s}}$ multidimensional space, being ℕ the set of the natural numbers, so that is possible to write:

(1) $$N\equiv \left\{N_{j}\right\}{\;}_{j=1}^{n_{s}}\in {\mathbb{N}}^{n_{s}}$$

Let us consider now a set of $n_{r}$ chemical reactions and their inverses:

(2) $$s_{1}^{\left(r\right)}X_{1}+s_{2}^{\left(r\right)}X_{2}+\ldots \to l_{1}^{\left(r\right)}X_{1}+l_{2}^{\left(r\right)}X_{2}+\ldots \;\;r=1,2,\ldots ,n_{r}$$

(3) $$l_{1}^{\left(r\right)}X_{1}+l_{2}^{\left(r\right)}X_{2}+\ldots \to s_{1}^{\left(r\right)}X_{1}+s_{2}^{\left(r\right)}X_{2}+\ldots \;\;r=n_{r}+1,\ldots 2n_{r}$$

That specifies, in general, a chemical reaction network. The coefficients of Equations (2) and (3) i.e., $\left\{s_{j}^{\left(r\right)},\;l_{j}^{\left(r\right)}\right\}$ for a given reaction r are known as the stoichiometric coefficients of the reaction. In general, each reaction of an arbitrary type r that takes place inside the tank, change the state of the system, which moves from the point $N\in {\mathbb{N}}^{n_{s}}$ to the point $N^{\prime }\in {\mathbb{N}}^{n_{s}}$ given by:

(4) $$N\equiv \left\{N_{j}\right\}{\;}_{j=1}^{n_{s}}\in {\mathbb{N}}^{n_{s}}\to N^{\prime }\equiv \left\{N_{j}+l_{j}^{\left(r\right)}-s_{j}^{\left(r\right)}\right\}=N+\nu^{\left(r\right)},$$

where in Equation (4), the vector $\nu^{\left(r\right)}={\left\{l_{j}^{\left(r\right)}-s_{j}^{\left(r\right)}\right\}}_{j=1}^{n_{s}}$ defines the changes in the mixture state, which take place when one chemical reaction of the *r*-th type occurs inside the system volume Ω. If the inverse reaction of *r* happens inside the system then the final state is $N^{\prime }=N-\nu^{\left(r\right)}.$ Now, if we denote by $\xi_{r}$ the number of reactions of the *r*-th type that take place inside the system during a given time interval $\left[t_{0},t=t_{0}+\Delta t\right]$, and by ${\dot{\xi }}_{r}$ the reaction rate. Then, it follows that if at time $t_{0}$, the system state was $N_{0}$, then the state of the system at time $t=t_{0}+\Delta t$ will be given by the following expression:

(5) $$N^{\prime }=N_{0}+\sum_{r=1}^{2n_{r}}\xi_{r}\;\nu^{\left(r\right)}$$

The total number of reactions that can take place inside a closed tank is limited by the available population of each species of atoms, denoted by the superscript α. Then if the symbol $n_{j}^{\left(\alpha \right)}$ denotes the number of atoms of the α type contained in the chemical compound $X_{j},$ then the conservation of the number of atoms of a given element in any chemical reaction gives the following set of conservation laws:

(6) $$\sum_{j=1}^{n_{s}}l_{j}^{\left(r\right)}n_{j}^{\left(\alpha \right)}=\sum_{j=1}^{n_{s}}s_{j}^{\left(r\right)}n_{j}^{\left(\alpha \right)}\;\;\;\mathrm{for}\;\alpha =1,2,\ldots .,n_{\alpha \tau }\;\mathrm{and}\;r=1,2,\ldots ,2n_{r},$$

being $n_{\alpha \tau }$ the total number of different elements contained in the tank. Next, Equation (6) can be rewritten as follows:

(7) $$\sum_{j=1}^{n_{s}}(l_{j}^{\left(r\right)}-s_{j}^{\left(r\right)})n_{j}^{\left(\alpha \right)}=\nu^{\left(r\right)}\cdot n^{\left(\alpha \right)}=0\;\;\mathrm{for}\;\alpha =1,2,\ldots ,n_{\alpha \tau }\;\mathrm{and}\;r=1,2,\ldots .2n_{r},$$

where $\nu^{\left(r\right)}\cdot n^{\left(\alpha \right)}$ denotes the scalar product in ${\mathbb{N}}^{n_{s}}.$ Equation (7) is true for all the reactions types and all the kinds of atoms.

Because the number of atoms of each element inside the tank is conserved, then in a closed system we can express this fact as follows:

(8) $$N\left(t\right)\cdot n^{\left(\alpha \right)}=N_{0}^{\left(\alpha \right)}\;\mathrm{for}\;\alpha =1,2,\ldots ,n_{\alpha \tau },$$

where $N\left(t\right)\equiv \left(N_{1}\left(t\right),\;N_{2}\left(t\right),\ldots ,N_{n_{s}}\left(t\right)\right)$, is the number of molecules of each chemical compound inside the tank at a given time t, and $N_{0}^{\left(\alpha \right)}$ is the total number of atoms of the element α, which are available initially at time *t* = 0. If the system is open this number can change with time and the conservation law (8) transform in:

(9) $$N\left(t\right)\cdot n^{\left(\alpha \right)}=N_{o}^{\left(\alpha \right)}\left(t\right)$$

Equation (9) expresses the fact that the number of atoms of each element can change with time in an open chemical system in which specific types of molecules can be injected or extracted along the time.

Equations (8) and (9) define a set of $n_{\alpha \tau }$ hyperplanes in the space ${\mathbb{N}}^{n_{s}}$. The part of the space ${\mathbb{N}}^{n_{s}},$ which is accessible starting from the initial point $N_{0}$ for a closed system is in the intersection of the hyperplanes defined by Equation (8). Notice that not all points are accessible due to the restrictions imposed by the finite number of atoms of each element.

The degree of advance $d\xi_{r}$ of a chemical reaction r during an infinitesimal time interval dt can be expressed in terms of the change in the number of molecules of the chemical compounds as follows [35]:

(10) $$\frac{dN_{1}^{\left(r\right)}}{l_{1}^{\left(r\right)}-s_{1}^{\left(r\right)}}=\frac{dN_{2}^{\left(r\right)}}{l_{2}^{\left(r\right)}-s_{2}^{\left(r\right)}}=\ldots =d\xi_{r}$$

Therefore, the change $dN_{j}^{\left(r\right)}$ in the number of molecules of the chemical compound $X_{j}$, produced by a reaction r with degree of advance $d\xi_{r}$ is obviously given, because of Equation (10), by:

(11) $$dN_{j}^{\left(r\right)}=\nu_{j}^{\left(r\right)}d\xi_{r}$$

From the Gibbs relation, the total change in the system entropy produced by the reactions taking place in the tank with volume Ω, when not external exchange of the chemical compounds (closed system) occurs, is given by the expression:

(12) $$TdS=dU-p\;d\Omega -\sum_{r=1}^{2n_{r}}\sum_{j=1}^{n_{S}}\mu_{j\;}dN_{j}^{\left(r\right)},$$

where $\mu_{j}$ is the chemical potential for the compound $X_{j}$, and $dS=d_{i}S+d_{e}S$, is the sum of the internal and external entropy changes. Notice that the external change can be produced by external exchanges of chemical compounds and/or energy. Using Equation (12) in Equation (11) and defining the chemical affinity for the *r*-th reaction in the form:

(13) $$A^{\left(r\right)}=-\overset{n_{s}}{\underset{j=1}{\sum }}\nu_{j}^{\left(r\right)}\mu_{j}$$

It is obtained the rate of change of the entropy with time:

(14) $$\frac{dS}{dt}=\frac{1}{T}\frac{dU}{dt}-\frac{p}{T}\frac{\;d\Omega }{dt}+\frac{1}{T}\overset{2n_{r}}{\underset{r=1}{\sum }}A^{\left(r\right)}{\dot{\xi }}_{r}$$

For non-equilibrium system thermodynamics processes, it is important to know their thermodynamic fluxes and their generalized thermodynamic forces. To do this, we consider the rate of change of the entropy per unit volume in an isolated system with constant volume and constant internal energy. This production rate of entropy denoted by σ, is given because of Equation (14) by:

(15) $$\sigma ={\left(\frac{1}{\Omega }\frac{dS}{dt}\right)}_{U,\Omega }=\frac{1}{T}\overset{2n_{r}}{\underset{r=1}{\sum }}A^{\left(r\right)}\frac{1}{\Omega }\;{\dot{\xi }}_{r}=\frac{1}{T}\overset{2n_{r}}{\underset{r=1}{\sum }}A^{\left(r\right)}J^{\left(r\right)}$$

From Equation (15), it is obtained that thermodynamic forces are the affinities and the thermodynamic fluxes are the reaction rates per unit volume, which are given because of Equation (15) by:

(16) $$J^{\left(r\right)}=\frac{1}{\Omega }\;{\dot{\xi }}_{r}$$

If the tank is well stirred, we can consider the system as homogeneous because matter flux of chemical compounds produced by the system in-homogeneities does not exist. Because of the thermodynamic fluxes depend, in first order approximation, linearly on the affinities i.e., $J^{\left(r\right)}=\sum_{r=1}^{2n_{r}}L_{r,s}A^{\left(s\right)}$, then it follows that at chemical equilibrium (σ=0), i.e., for not evolution of all the chemical reactions inside the system we must have:

(17) $$A^{\left(r\right)}=0\;for\;r=1,2\ldots 2n_{r}$$

From conditions (17) and the expression for the chemical potentials given by [35,36,37]:

(18) $$\mu_{j}\left(p,T,N_{j}\right)=\mu_{j}^{\left(0\right)}\left(p,T\right)+RTlog\left(a_{j}\right),$$

where, $a_{j}$ is the chemical activity of the *j*-th component and $\mu_{j}^{\left(0\right)}\left(p,T\right)$ is the chemical potential of the compound in a pure substance. Then, it is easily deduced because of Equation (13) the mass action law for the direct chemical reaction:

(19) $${\bar{a}}_{1}^{l_{1}^{\left(r\right)}-s_{1}^{\left(r\right)}}{\bar{a}}_{2}^{l_{2}^{\left(r\right)}-s_{2}^{\left(r\right)}}\ldots {\bar{a}}_{n_{s}}^{l_{n_{s}}^{\left(r\right)}-s_{n_{s}}^{\left(r\right)}}=K^{\left(r\right)}\left(p,T\right),$$

where $K^{\left(r\right)}\left(p,T\right)$ is the equilibrium constant for the reaction *r*, at given pressure *p* and temperature *T*, being ${\bar{a}}_{1,\;}{\bar{a}}_{2},\ldots ,{\bar{a}}_{n_{s}}$ the activities of the chemical compounds at equilibrium conditions denoted by the overbar. From Equations (13), (18) and (19), it is deduced that the equilibrium constant is given by the expression:

(20) $$K^{\left(r\right)}\left(p,T\right)=\exp\left\{\overset{n_{s}}{\underset{j=1}{\sum }}\nu_{j}^{\left(r\right)}\mu_{j}^{\left(0\right)}\left(T,p\right)/\left(RT\right)\right\}$$

It is important to realize that $a_{j}=\gamma_{j}x_{j}$ are the chemical activities for non-ideal systems, being $\gamma_{j}$ the activity coefficients, and $x_{j}$ the molar fractions of the chemical compounds.

### 2.2. Probability Distribution Function (PDF) at Equilibrium 

In this section we study the PDF of the number of molecules of each chemical compound at equilibrium conditions. Einstein was the first to invert the classical Boltzmann relation between entropy and probability, obtaining the probability in terms of the system entropy [4,38]. Let us consider a system that depends on a set of thermodynamic magnitudes as the internal energy U, the volume Ω and the number of molecules $N_{j}$ of each chemical compound $X_{j}$, in this case we can write $S=S\left(U,\Omega ,N_{1},N_{2},\ldots ,N_{n_{s}}\right).$ In addition, according to the second principle at equilibrium conditions, the variables take the values that maximize the system entropy, and using statistical mechanics it is possible to obtain the probability of fluctuations of the variables around the maximum entropy state, described by $\bar{S}=S\left(\bar{U},\bar{\Omega },{\bar{N}}_{1},{\bar{N}}_{2},\ldots {\bar{N}}_{n_{s}}\right)$. If we assume that the tank volume remains constant, then from Einstein relation, we can write:

(21) $$P\left(U,N_{1},N_{2},\ldots N_{n_{s}}\right)=\bar{P}\exp\left\{\frac{S\left(U,N_{1},N_{2},\ldots N_{n_{s}}\right)}{k}-\frac{\bar{S}\;\;\left(\bar{U},{\bar{N}}_{1},{\bar{N}}_{2},\ldots {\bar{N}}_{n_{s}}\right)}{k}\right\}=\bar{P}\exp\left(\frac{\Delta S}{K}\right),$$

being *k* the Boltzmann constant, and $\bar{P}$ the probability at equilibrium conditions. Next, we use the Gibbs relation (12) at constant U and Ω, that yields, if the system is closed:

(22) $${\left(TdS\right)}_{U,\Omega }=-\sum_{r=1}^{2n_{r}}\sum_{j=1}^{n_{S}}\mu_{j\;}dN_{j}^{\left(r\right)}=-\sum_{j=1}^{n_{S}}\mu_{j\;}dN_{j},$$

where $dN_{j}=\sum_{r=1}^{2n_{r}}dN_{j}^{\left(r\right)}$ is the change in the number of molecules belonging to *j*-th species produced by all the reactions occurring inside the stirred tank. Expanding the entropy in Taylor series up to second order around the maximum entropy state, it is found that:

(23) $${\left(\Delta S\right)}_{U,\Omega }=\overset{n_{s}}{\underset{i,j=1}{\sum }}\frac{1}{2}{\left(\frac{\partial^{2}S}{\partial N_{i}\partial N_{j}}\right)}_{U,\Omega ,\bar{N}}\left(N_{i}-{\bar{N}}_{i}\right)\left(N_{j}-{\bar{N}}_{j}\right)$$

Next from the Gibbs relation it is deduced that the chemical potential is given by:

(24) $$\mu_{i}=-T{\left(\frac{\partial S}{\partial N_{i}}\right)}_{U,\Omega ,N_{j\neq i}}$$

Finally, from Equations (21), (23) and (24), it is obtained that the probability of fluctuations in the number of chemical compounds around the equilibrium state is given by:

(25) $$P=\bar{P}\exp\left(-\frac{1}{2kT}\overset{n_{s}}{\underset{i,j=1}{\sum }}{\left(\frac{\partial \mu_{i}}{\partial N_{j}}\right)}_{U,\Omega ,\bar{N}}\left(N_{i}-{\bar{N}}_{i}\right)\left(N_{j}-{\bar{N}}_{j}\right)\right)$$

### 2.3. Roots of Chemical Reaction Kinetics 

We start studying the case of the reaction rate of two different chemical compounds, $X_{i}$ and $X_{j},$ which can react producing a chemical compound, $X_{k}$. If we have at time *t*, $N_{i}$ and $N_{j}$ molecules of these chemical compounds inside the tank with volume Ω and temperature *T*, and we denote by $\sigma_{ij}$ the collision cross section of both molecules, and by ${\vec{v}}_{r}={\vec{v}}_{i}-{\vec{v}}_{j}$ their relative velocity. Then, if the velocity of the individual particles follows a Maxwell-Boltzmann distribution, it is easy to prove that the relative velocity of the particles follows also a Maxwell-Boltzmann distribution, which has the form:

(26) $$f_{B,ij}\left(v_{r}\right)d^{3}v_{r}={\left(\frac{m}{2\pi kT}\right)}^{3/2}\exp\left\{-\frac{mv_{r}^{2}}{2kT}\right\}d^{3}v_{r},$$

where $\varepsilon_{CM}=\frac{1}{2}mv_{r}^{2}$ is the center of mass (CM) energy of both particles, being $m=\frac{m_{i}m_{j}}{m_{i}+m_{j}}$ the reduced mass. To produce the reaction, we need at least a minimum threshold energy in the CM system denoted by $\varepsilon_{CM,0}=\frac{1}{2}mv_{r,0}^{2}$, so that, if the CM energy of the colliding compounds is smaller than $\varepsilon_{CM,0}$, then the reaction does not take place. So, if the tank is well stirred and at temperature *T* then we can compute the collision $R_{c,ij}$ and reaction rates $R_{r,ij}$ per unit volume by means of the following expressions:

(27) $$R_{c,ij}=\int_{0}^{\infty }dv_{r}\int_{0}^{\pi }v_{r}\sigma_{col,ij}f_{B,ij}\left(v_{r}\right)v_{r}^{2}2\pi \sin\theta d\theta \frac{N_{i}}{\Omega }\frac{N_{j}}{\Omega }$$

where we have considered that $d^{3}v_{r}=v_{r}^{2}dv_{r}d\Omega_{solid}=v_{r}^{2}dv_{r}2\pi \sin\theta d\theta$. Substituting Equation (26) into Equation (27) and performing the integrals in Equation (27), (see the Handbook of Mathematical Functions section 7 on Error functions and Fresnel integrals [39]), yields after some calculus:

(28) $$R_{c,ij}=\sigma_{col,ij}{\left(\frac{8\pi kT}{m}\right)}^{1/2}\frac{N_{i}}{\Omega }\frac{N_{j}}{\Omega }=\sigma_{col,ij}{\bar{v}}_{r,ij}\frac{N_{i}}{\Omega }\frac{N_{j}}{\Omega },$$

where, ${\bar{v}}_{r,ij}={\left(\frac{8\pi kT}{m}\right)}^{1/2}$ is the average relative velocity between the molecules at temperature *T*. But not all the collisions between the molecules, $X_{i}$ and $X_{j}$ produce reactions $X_{i}+X_{j}\to X_{k}$. Because only those collisions with energy in the center of mass system bigger than the critical energy will yield reactions. Therefore, to obtain the reaction rate per unit volume one must consider the lower limit of the velocity in the integral of Equation (27) to be equal to the velocity corresponding to the critical energy. In addition, according to Gillespie [6], the reaction will take place only if the collisional point of contact takes place inside the sensitive area of each colliding molecule, this fact introduces an additional factor, that will be denoted by $p_{i,j}^{\left(s\right)}$. This factor will depend on the subtended solid angle of the sensitive area for each molecule. So, the reaction rate per unit volume for bimolecular reactions will be given by:

(29) $$\begin{array}{c} R_{r,ij}=\int_{v_{r,0}}^{\infty }dv_{r}\int_{0}^{\pi }v_{r}\sigma_{col,ij}f_{B,ij}\left(v_{r}\right)v_{r}^{2}2\pi sin\theta d\theta \frac{N_{i}}{\Omega }\frac{N_{j}}{\Omega }p_{i,j}^{\left(s\right)}\\ \;\;\;\;\;\;\;\;=\sigma_{col,ij}{\bar{v}}_{r,ij}\frac{N_{i}}{\Omega }\frac{N_{j}}{\Omega }\left(1+\frac{\varepsilon_{CM,0}}{kT}\right)\exp\left(-\frac{\varepsilon_{CM,0}}{kT}\right)p_{i,j}^{\left(s\right)}, \end{array}$$

where $p_{i,j}^{\left(s\right)}$ is given by the expression:

(30) $$p_{ij}^{\left(s\right)}=\frac{\omega_{i}^{\left(s\right)}\omega_{j}^{\left(s\right)}}{{\left(4\pi \right)}^{2}},$$

being $\omega_{i}^{\left(s\right)}$ and $\omega_{j}^{\left(s\right)}$ the solid angles which are subtended by the reaction sensitive areas of molecules *i* and *j* respectively.

So, the reaction rate for bimolecular collisions can be expressed because of Equations (28) and (29) as follows:

(31) $$R_{r,ij}=R_{c,ij}\left(1+\frac{\varepsilon_{CM,0}}{kT}\right)\exp\left(-\frac{\varepsilon_{CM,0}}{kT}\right)p_{ij}^{\left(s\right)}=R_{c,ij}\eta_{ij},$$

where $\eta_{ij}$ is the probability that one collision between two chemical compounds $X_{i}$ and $X_{j}$ yields one reaction event, this probability is given by the expression:

(32) $$\eta_{ij}=\left(1+\frac{\varepsilon_{CM,0}}{kT}\right)\exp\left(-\frac{\varepsilon_{CM,0}}{kT}\right)p_{ij}^{\left(s\right)}$$

The propensity function $\gamma_{r}\left(N\right)$ for this type of bimolecular reaction *r* is defined in such a way that $\gamma_{r}\left(N\right)dt$ gives the probability to have one reaction event of type *r* in the time interval between (t,t+dt) inside the stirred tank of volume Ω assuming that the system was at time t in the state characterized by the state vector N. On account of this definition and expression (31), we can compute the propensity for a bimolecular reaction of different species as:

(33) $$\gamma_{r}\left(N\right)dt=\Omega \;R_{c,ij}\eta_{ij}=K_{r}\frac{1}{\Omega }N_{i}N_{j}dt\;\mathrm{with}\;j\neq i\;\mathrm{and}\;K_{r}=\sigma_{col,ij}{\bar{v}}_{r,ij}(1+\;\frac{\varepsilon_{CM,0}}{kT})\exp\left(-\frac{\varepsilon_{CM,0}}{kT}\right)p_{ij}^{\left(s\right)}$$

Therefore $K_{r}dt$ gives the probability to have one reaction of type *r* between t and t+dt per i,j pair and per unit volume. Notice that the total number of i,j couples per unit volume is $\frac{N_{i}N_{j}}{\Omega }$.

If the chemical compounds that collide are identical then, $m_{i}=m_{j}$, and the number of different couples that can collide inside Ω is $N_{i}\left(N_{i}-1\right)/2$, then proceeding as previously it is obtained that the collision rate per unit volume is:

(34) $$R_{c,ii}=\sigma_{col,ii}{\left(\frac{8\pi kT}{m}\right)}^{1/2}\frac{N_{i}}{2\;\Omega }\frac{\left(N_{i}-1\right)}{\Omega }=\sigma_{col,ij}{\bar{v}}_{r,ii}\frac{N_{i}}{2\;\Omega }\frac{\left(N_{i}-1\right)}{\Omega },$$

where $\sigma_{col,ii}$ denotes the collision cross-section between two identical molecules, $m=\frac{m_{i}}{2}$ is the reduced mass and ${\bar{v}}_{r,ii}$ denotes the average relative velocity between two identical particles. The rate of collisions per unit volume with energy in the CM system bigger than the critical energy and that produce reactions $X_{i}+X_{i}\to X_{l}$ is:

(35) $$R_{r,ii}=R_{c,ii}\left(1+\frac{\varepsilon_{CM,0}}{kT}\right)\exp\left(-\frac{\varepsilon_{CM,0}}{kT}\right)p_{ii}^{\left(s\right)}=R_{c,ii}\eta_{ii},$$

where the expression for $\eta_{ii}$ is given by Equation (32). The probability to have one reaction $X_{i}+X_{i}\to X_{l}$ in the differential interval (t, t+dt), if the system was at the state N at time *t*, is given on account of Equations (34) and (35) by:

(36) $$\gamma_{r}\left(N\right)dt=\Omega \;R_{c,ii}\eta_{ii}=K_{r}\frac{N_{i}\left(N_{i}-1\right)}{2\Omega }dt,$$

where $\gamma_{r}\left(N\right)$ denotes the propensity for this chemical reaction, and $K_{r}dt$ gives the probability to have one reaction in the tank per couple of identical chemical compounds per unit volume.

Generalizing these previous expressions to a generic reaction *r*: $\sum_{j=1}^{n_{s}}s_{j}^{\left(r\right)}\to \sum_{i=1}^{n_{s}}l_{j}^{\left(r\right)}$ the propensity can be expressed by the following equation:

(37) $$\gamma_{r}\left(\vec{N}\right)=K_{r}\Omega \;\overset{n_{s}}{\underset{j=1}{\prod }}\Omega^{-s_{j}^{\left(r\right)}}\left(\begin{array}{c} N_{j}\\ s_{j}^{\left(r\right)} \end{array}\right).$$

For a bimolecular reaction of identical particles, we have that $s_{j}^{\left(r\right)}=2$, and Equation (37) yields Equation (36) for this case. For a bimolecular reaction of different chemical compounds $X_{i}$, and $X_{j}$ $s_{i}^{\left(r\right)}=1$ and $s_{j}^{\left(r\right)}=1,$ and Equation (37) gives for this case Equation (33).

### 2.4. The Chemical Master Equation

Let us consider the set of reactions $\sum_{j}^{n_{s}}s_{j}^{\left(r\right)}\to \sum_{j}^{n_{s}}l_{j}^{\left(r\right)}$, with $n_{r}=1,2,\ldots ,\;n_{r},$ with kinetic constants denoted by $K_{r}$ and their inverses ranging from $r=n_{r+1}+1,\ldots ,\;2n_{r}$ with kinetic constants denoted by $K_{n_{r}+1}=K_{1}^{\prime }$, $K_{n_{r}+2}=K_{2}^{\prime }$, …$K_{2n_{r}}=K_{n_{r}}^{\prime }$. We notice that the change vectors $\nu^{\left(r\right)}$ of the population of the different compounds produced by the inverse reactions are related to their corresponding change vectors of the direct ones by the set of equations:

(38) $$\nu^{\left(n_{r}+1\right)}=-\nu^{\left(1\right)};\;\nu^{\left(n_{r}+2\right)}=-\nu^{\left(2\right)};\ldots \nu^{\left(2n_{r}\right)}=-\nu^{\left(n_{r}\right)}$$

Next one defines a continuous time Markov process {N(t), t≥0} with discrete state space $N\left(t\right)\in {\mathbb{N}}^{n_{s}}$. The main characteristic of these processes is that the system state depends only on the last previously known state and not on the previous ones at earlier times. Therefore, the *n*-times conditional probability for a Markov process reduces to [40]:

(39) $$P_{n}\left(N_{n},t_{n}|N_{n-1},t_{n-1};\ldots ;N_{1},t_{1}\right)=P_{2}\left(N_{n},t_{n}|N_{n-1},t_{n-1}\right)\;\forall t_{n}\geq t_{n-1}\geq \ldots \geq t_{1}$$

The following equation, known as the Chapmann-Kolmogorov equation is true for all the Markov processes [40], and it is easily deduced because of the definition of conditional probability:

(40) $$P_{2}\left(N,t|N_{0},t_{0}\right)=\underset{N^{\prime }}{\sum }P_{2}\left(N,t|N^{\prime },t^{\prime }\right)P_{2}\left(N^{\prime },t^{\prime }|N_{0},t_{0}\right),$$

where the summation extend over all possible states $N^{\prime }$ at an intermediate time $t^{\prime }.$ To deduce the Master equation one consider a time instant $t^{\prime }=t-\Delta t$, arbitrarily close to *t*, and denoting by $T_{t}(N|N^{\prime })\geq 0$ the transition probability per unit time from the state $N^{\prime }$ to the state N at time *t*, then it is possible to write down, dropping the sub index 2 in the probability:

(41) $$P\left(N,t|N^{\prime },t-\Delta t\right)=T_{t}(N|N^{\prime })\Delta t+o\left(\Delta t\right)\;\mathrm{when}\;N^{\prime }\neq N$$

(42) $$P\left(N,t|N,t-\Delta t\right)=1-\sum_{N^{\prime }\neq N}T_{t}(N|N^{\prime })\Delta t\;+\;o\left(\Delta t\right),$$

where o(Δt) means that there are terms that goes to zero faster than Δt, and therefore for small Δt can be neglected. Because of Equations (41) and (42) in Equation (40), it is obtained:

(43) $$\begin{array}{ll} P\left(N,t|N_{0},t_{0}\right)= & \underset{N^{\prime }\neq N}{\sum }T_{t}(N|N^{\prime })\Delta tP\left(N^{\prime },t-\Delta t|N_{0},t_{0}\right)\\ & +\left(1-\underset{N^{\prime }\neq N}{\sum }T_{t}(N|N^{\prime })\Delta t\right)P\left(N,t-\Delta t|N_{0},t_{0}\right)+o\left(\Delta t\right) \end{array}$$

After some calculus, expanding $P\left(N,t-\Delta t|N_{0},t_{0}\right)$ and $P\left(N^{\prime },t-\Delta t|N_{0},t_{0}\right)$ in Taylor series and retaining only the terms of order Δt it is obtained:

(44) $$\partial_{t}P\left(N,t|N_{0},t_{0}\right)=\underset{N^{´\prime }}{\sum }\left[T_{t}(N|N^{\prime })P\left(N^{\prime },t^{\prime }|N_{0},t_{0}\right)-T_{t}(N^{\prime }|N)P\left(N,t|N_{0},t_{0}\right)\right]$$

Equation (44) is known as the Master equation, the first term of this equation gives the increase in the probability per unit time due to the transitions from other states $N^{\prime }$ to the state N at time *t*, while the second term gives the probability diminishing rate due to the transitions from the state N to other states $N^{\prime }$.

Let us discuss now the usual form of the Master Equation for chemical kinetics known as the chemical master equation (CME). This equation can be obtained directly from Equation (44), but it is more instructive to deduce it by a probability balance method. 

Let us write first a probability balance for $P\left(N,t+\Delta t|N_{0},t_{0}\right)$ in terms of the probability at an earlier time t, and the propensities using the propensities defined in the previous section. Because of the propensities only depend on the system collective state N when the tank system it is well stirred and the properties like the temperature are uniforms. Then, we can write because of the propensity definition and the change $\nu^{\left(r\right)}$ produced by each reaction type r on the number of molecules of each chemical compound:

(45) $$\begin{array}{l} P\left(N,t+\Delta t|N_{0},t_{0}\right)\\ \;\;\;\;\;\;\;\;=\overset{2n_{r}}{\underset{r=1}{\sum }}\left(\gamma_{r}\left(N-\nu^{\left(r\right)}\right)\Delta t+o\left(\Delta t\right)\right)P\left(N-\nu^{\left(r\right)},t|N_{0},t_{0}\right)\\ \;\;\;\;\;\;\;\;+\left\{1-\overset{2n_{r}}{\underset{r=1}{\sum }}\left(\gamma_{r}\left(N\right)\Delta t+o\left(\Delta t\right)\right)\right\}P\left(N,t|N_{0},t_{0}\right) \end{array}$$

Passing $P\left(N,t|N_{0},t_{0}\right)$ to the left hand side, dividing then both sides by Δt, and computing the limit when Δt→0, it is obtained, on account that $\underset{\Delta t\to 0}{\lim}o\left(\Delta t\right)/\Delta t\to 0$, the following equation for P(N,t), where we have omitted the $N_{0},t_{0}$ because we consider only those states that can be attained from the chemical system initial conditions:

(46) $$\partial_{t}P\left(N,t\right)=\overset{2n_{r}}{\underset{r=1}{\sum }}\left[\gamma_{r}\left(N-\nu^{\left(r\right)}\right)P\left(N-\nu^{\left(r\right)},t\right)-\gamma_{r}\left(N\right)P\left(N,t\right)\right]$$

This is the Chemical Master Equation that expresses the probability evolution of P(N,t) in terms of the propensities for each network reaction type and the probabilities $P\left(N-\nu^{\left(r\right)},t\right)$ to be at the states $N-\nu^{\left(r\right)}$ that can reach the state N in one reaction. The last term of this equation gives the probability to be removed from the state N by the different kinds of reactions.

Now we consider the following identity that holds for the inverse reactions:

(47) $$\nu^{\left(n_{r}+i\right)}=-\nu^{\left(i\right)}\;for\;i=1,2,\ldots ,n_{r}$$

Because of Equation (47), the CME (46) can be written in the following form:

(48) $$\begin{array}{l} \partial_{t}P\left(N,t\right)=\overset{n_{r}}{\underset{r=1}{\sum }}\left[\gamma_{r}\left(N-\nu^{\left(r\right)}\right)P\left(N-\nu^{\left(r\right)},t\right)-\gamma_{r}\left(N\right)P\left(N,t\right)\right]\\ \;\;\;\;\;\;\;\;+\overset{2n_{r}}{\underset{r=n_{r}+1}{\sum }}\left[\gamma_{r}\left(N+\nu^{\left(r-n_{r}\right)}\right)P\left(N+\nu^{\left(r-n_{r}\right)},t\right)-\gamma_{r}\left(N\right)P\left(N,t\right)\right] \end{array}$$

This is an alternative way of writing the CME because of the existing relations (47) between the direct and the inverse reactions.

### 2.5. Limitations of the Chemical Master Equation

To finish this section, we will discuss deeply the limitations of the CME formalism developed in the previous sections. As pointed out by Nicolis and Prigogine [4], the fact that the propensities depend on system collective variables implies that important fluctuation phenomena associated to local parameters of the system, as for instance local variations of the concentration, and correlation lengths over which two part of the system influence mutually are not considered in the approach of the CME. Also, Gillespie et al. [41], have discussed the conditions for validity of the CME in diffusion-limited systems, especially when bimolecular reactions occur, the two fundamental conditions which must hold are that the reactant molecules in the reacting system are at the same time “dilute” and “well-mixed”. These authors have proved that the satisfaction of both conditions is necessary for the predictions of the CME to be true in a system where bimolecular reactions take place. Notice that the requirement for diluteness in a solution of reacting chemical compounds applies only to the reactant molecules (solute), and not to the chemically inert solvent molecules as pointed out by Gillespie et al. [41]. However spatial effects are present in many chemical and biological systems, and therefore for these systems do not hold the assumption of spatially uniform or well mixed distribution of chemical reactants. In his seminal paper, Turing [42,43] demonstrated, theoretically, that a system of reacting and diffusing chemicals compounds could spontaneously evolve to spatially heterogeneous patterns when departing from an initially uniform state in response to very small perturbations. He considered a very simple chemical system formed by two chemical compounds, in which one was an activator and the other one an inhibitor. That is, one chemical compound, the activator, stimulated and enhanced the production of the other compound, the inhibitor, which, in turn, depleted or inhibited the formation of the activator. Turing showed that if the diffusion of the inhibitor was bigger than that of the activator, then a diffusion-driven instability could result. This is a non-intuitive behavior, because we are used to think that diffusion is a homogenizing process. The first experimental evidence of Turing spatial patterns was observed in the chlorite-iodide-malonic acid (CIMA) reaction with starch [43]. More recently Maini, Painter and Chau [44], have reviewed the formation of spatial patterns in chemical and biological systems. 

In this way spatial stochastic effects, which it is well known play important roles in many systems are neglected in the CME approach. The situation for these cases can be amended using the Reaction Diffusion Master Equation (RDME), in which space Ω is partitioned discretely into an arbitrary number of voxel (Volume Elements) each one with a volume ΔΩ [44,45]. Diffusion can then occur between different voxels, and reactions can occur within voxels on the assumption that reactants are well-mixed. 

However, even in this case the description is not complete for many system because if the reactants are dissolved in a solvent, the motion of the solvent affects to the reactants, and we can have in addition to the normal diffusion process, the one known as turbulent diffusion produced by the small eddies and also it is possible to have transport due to the solvent motion. 

It is important to know the factors which influence the spatial correlations to understand the limitations of the CME, consider for instance a simple example of non-equilibrium non-linear chemical kinetics to understand the correlation origin. Nicolis, Prigogine, Van Kampen, and Van Den Broeck among others started the study of this issue [4,8,46]. Consider the following chemical reaction $A\overset{K_{1}^{\left(+\right)}}{\to }2X$, and its inverse $2X\overset{K_{1}^{\left(-\right)}}{\to }A$. For this simple system the particles are created in couples of spatial and time correlated particles by the reaction $A\overset{K_{1}^{\left(+\right)}}{\to }2X$, while for the inverse reaction $2X\overset{K_{1}^{\left(-\right)}}{\to }A$ between particle pairs which are close enough this reaction depletes the system from pairs of X particles and produce A molecules. Because the particles *X* created in the same reaction are both produced at the same spatial point $\vec{r}$ and time t, then these particles are obviously correlated. Then, due to diffusion this correlation propagates spatially until one of the two particles suffers a reaction, if this reaction takes place on average at a rate κ, then the characteristic correlation length, denoted by $l_{c}$ is given by the expression [8,46]:

(49) $$l_{c}={\left(\frac{D_{F}}{\kappa }\right)}^{1/2},$$

being $D_{F}$, the diffusion coefficient of the chemical species *X*. Partitioning the space into equal volume cubic cells with volume ΔΩ, and denoting the centers of this cells by their position vectors $\vec{r}$, and by $N_{\vec{r}}^{\left(X\right)}\left(t\right)$ the number of molecules of the species *X* contained in each sub-volume at time *t*. Then it is possible to deduce the multivariate chemical master equation (MCME) obeyed by the probability P$\left(\left\{N_{\vec{r}}^{\left(X\right)}\left(t\right)\right\},t\right)$, where the symbol $\left\{N_{\vec{r}}^{\left(X\right)}\left(t\right)\right\}$ denotes all $N_{\vec{r}}^{\left(X\right)}\left(t\right)$ values $\forall \vec{r}$. The time evolution of this multivariate probability is produced by two different mechanisms the first one are the chemical reactions occurring inside the cells, and the second one is the diffusion of particles between adjacent cells [8,46]. The equal time correlation function $g^{\left(X\right)}\left(\vec{r},{\vec{r}}^{\prime },t\right)$ at two different point $\vec{r}\neq {\vec{r}}^{\prime }\;\mathrm{at}\;\mathrm{the}\;\mathrm{same}\;\mathrm{time}\;\mathrm{instant}\;\mathrm{is}$ defined in terms of fluctuations $\delta \rho_{\vec{r}}^{\left(X\right)}$ of the intensive variable ρr→(X)=Nr→(X)ΔΩ, evaluated at these points in the continuous limit when ΔΩ→0, as follows:

(50) $$g^{\left(X\right)}\left(\vec{r},{\vec{r}}^{\prime },t\right)=\langle \delta \rho_{\vec{r}}^{\left(X\right)}\delta \rho_{{\vec{r}}^{\prime }}^{\left(X\right)}\rangle \;for\;\vec{r}\neq {\vec{r}}^{\prime }$$

The equation obeyed by the correlation function at two different points can be calculated by the usual procedure of multiplying the MCME by $N_{\vec{r}}^{\left(X\right)}\left(t\right)N_{{\vec{r}}^{\prime }}^{\left(X\right)}\left(t\right)$, followed by summation over all $\left\{N_{\vec{r}}^{\left(X\right)}\left(t\right)\right\}$. The steady state solution of the equation obeyed by the equal time correlation function is given by the following expression [8,46]:

(51) $$g^{\left(X\right)}\left(\vec{r},{\vec{r}}^{\prime },t\right)=\frac{J_{X}^{\left(1\right)}}{4\pi \left|\vec{r}-{\vec{r}}^{\prime }\right|D_{F}}\exp\left(-\frac{\left|\vec{r}-{\vec{r}}^{\prime }\right|}{l_{c}}\right)\;for\;\vec{r}\neq {\vec{r}}^{\prime }$$

where the characteristic correlation length $l_{c}$ is given by Equation (49), being κ the rate at which the *X* molecules are destroyed by the inverse reaction, this rate is obviously given for this simple example by:

(52) $$\kappa =K_{1}^{\left(-\right)}\langle \rho_{\vec{r}}^{\left(x\right)}\rangle {}^{2},$$

$\mathrm{and}\;J_{X}^{\left(1\right)}$ is the chemical thermodynamic flux for the *X* chemical compound which is given by:

(53) $$J_{X}^{\left(1\right)}=2K_{1}^{\left(+\right)}\rho^{\left(A\right)}-K_{1}^{\left(-\right)}\langle \rho_{\vec{r}}^{\left(X\right)}\rangle {}^{2}$$

If the system is at equilibrium conditions then the flux $J_{X}^{\left(1\right)}=0$, and there is not local creation of correlations. Also notice that according to Equation (49), if $K_{1}^{\left(-\right)}$ increases then the correlation length diminishes. Therefore, correlations can be important when the chemical system is far from equilibrium and therefore spatial effects can become important under this situation.

## 3. Methods to Obtain the Moments of the Probability Distribution in Chemical Reaction Networks

### 3.1. The Method of the Probability Generating Function or Z-Transform

The method known as the probability generating function or simply generating function method, or *z*-transform method consist in defining the *z*-transform of the function P(N,t) by means of the following expression [47]:

(54) $$G\left(z,t\right)=\overset{\infty }{\underset{N_{1}=0}{\sum }}\overset{\infty }{\underset{N_{2}=0}{\sum }}\ldots \overset{\infty }{\underset{N_{n_{s}=0}}{\sum }}z_{1}^{N_{1}}z_{2}^{N_{2}}\ldots z_{n_{s}}^{N_{n_{s}}}P\left(N_{1,}N_{2},\ldots ,N_{n_{s}},t\right)=\underset{N}{\sum }\overset{n_{s}}{\underset{j=1}{\prod }}z_{j}^{N_{j}}P(N,t),$$

where $z\equiv \left(z_{1},z_{2},\ldots ,z_{n_{s}}\right)$, denotes the corresponding points in the transformed space.

From the multivariate generating function G(z,t), is easy to obtain various kinds of moments. For instance, the *k*-th factorial-moment can be obtained easily from G(z,t) as follows:

(55) $$\langle N_{i}\left(N_{i}-1\right)\ldots \left(N_{i}-k+1\right)\rangle ={\left[\frac{\partial^{k}}{\partial z_{i}^{k}}\;G\left(z,t\right)\right]}_{z_{1}=\ldots =z_{n_{s}}=1}$$

The second order cross-moment $\langle N_{i}N_{j}\rangle$, can also be obtained easily from G(z,t):

(56) $$\langle N_{i}N_{j}\rangle ={\left[\frac{\partial^{2}}{\partial z_{i}\partial z_{j}}\;G\left(z,t\right)\right]}_{z_{1}=\ldots =z_{n_{s}}=1}$$

To obtain the evolution equation for the multivariate generating function we proceeds as follows, first we multiply the chemical master Equation (46), by $z_{1}^{N_{1}}z_{2}^{N_{2}}\ldots z_{n_{s}}^{N_{n_{s}}}$, and then we sum up for all the N values i.e., $N_{1}\;\mathrm{from}\;0\;\mathrm{to}\;\infty$, $N_{2}$ from 0 to ∞, and so on, and finally, we substitute the propensities by their expressions given by Equation (37). This set of operations yields the following result:

(57) $$\begin{array}{l} \partial_{t}G\left(z,t\right)=\overset{n_{r}}{\underset{r=1}{\sum }}\underset{N}{\sum }K_{r}^{\left(+\right)}\Omega \{\overset{n_{s}}{\underset{j=1}{\prod }}\left[\Omega^{-s_{j}^{\left(r\right)}}\left(\begin{array}{c} N_{j}-\nu_{j}^{\left(r\right)}\\ s_{j}^{\left(r\right)} \end{array}\right)z_{j}^{N_{j}}\right]P\left(N-\nu^{\left(r\right)},t\right)\\ \;\;\;\;\;\;\;\;-\overset{n_{s}}{\underset{j=1}{\prod }}\left[\Omega^{-s_{j}^{\left(r\right)}}\left(\begin{array}{c} N_{j}\\ s_{j}^{\left(r\right)} \end{array}\right)z_{j}^{N_{j}}\right]P\left(N,t\right)\}\\ \;\;\;\;\;\;\;\;+\overset{n_{r}}{\underset{r^{\prime }=1}{\sum }}\underset{N}{\sum }K_{r}^{\left(-\right)}\Omega \{\overset{n_{s}}{\underset{j=1}{\prod }}\left[\Omega^{-l_{j}^{\left(r^{\prime }\right)}}\left(\begin{array}{c} N_{j}-\nu_{j}^{\left(r^{\prime }\right)}\\ s_{j}^{\left(r^{\prime }\right)} \end{array}\right)z_{j}^{N_{j}}\right]P\left(N+\nu^{\left(r^{\prime }\right)},t\right)\\ \;\;\;\;\;\;\;\;-\overset{n_{s}}{\underset{j=1}{\prod }}\left[\Omega^{-l_{j}^{\left(r^{\prime }\right)}}\left(\begin{array}{c} N_{j}\\ s_{j}^{\left(r^{\prime }\right)} \end{array}\right)z_{j}^{N_{j}}\right]P\left(N,t\right)\} \end{array}$$

To obtain Equation (57), we have considered Equation (46), and we have defined $K_{r}^{\left(+\right)}$ which denotes the $K_{r}$ constant for the direct chemical reaction *r*, while $K_{r}^{\left(-\right)}$ denotes this constant for the inverse of the direct *r*-th reaction. Considering that all the components of $N^{\prime }=N-\nu^{\left(r\right)}$ are positive, because physically only can take positive values, then it follows that the probabilities $P\left(N-\nu^{\left(r\right)},t\right)$ must be zero when some component of $N^{\prime }=N-\nu^{\left(r\right)}$ is negative. Because the components of the vector $N^{\prime }$ can take only values between 0 and ∞, then we can perform a change of variables in the summations from N to $N^{\prime }$. Finally, we rename again $N^{\prime }$ as N. Notice than when performing these operations for a given reaction *r* we have that $z_{j}^{N_{j}}=z_{j}^{N_{j}-\nu_{j}^{\left(r\right)}+\nu_{j}^{\left(r\right)}}=z_{j}^{N_{j}^{\prime }+\nu_{j}^{\left(r\right)}},$ so finally, Equation (57) can be rewritten in the form:

(58) $$\begin{array}{l} \partial_{t}G\left(z,t\right)=\overset{n_{r}}{\underset{r=1}{\sum }}\underset{N}{\sum }K_{r}^{\left(+\right)}\Omega \{\overset{n_{s}}{\underset{j=1}{\prod }}\left[\Omega^{-s_{j}^{\left(r\right)}}\left(\begin{array}{c} N_{j}\\ s_{j}^{\left(r\right)} \end{array}\right)z_{j}^{N_{j}+\nu_{j}^{\left(r\right)}}\right]P\left(N,t\right)\\ \;\;\;\;\;\;\;\;-\overset{n_{s}}{\underset{j=1}{\prod }}\left[\Omega^{-s_{j}^{\left(r\right)}}\left(\begin{array}{c} N_{j}\\ s_{j}^{\left(r\right)} \end{array}\right)z_{j}^{N_{j}}\right]P\left(N,t\right)\}\\ \;\;\;\;\;\;\;\;+\overset{n_{r}}{\underset{r^{\prime }=1}{\sum }}\underset{N}{\sum }K_{r^{\prime }}^{-}\Omega \{\overset{n_{s}}{\underset{j=1}{\prod }}\left[\Omega^{-l_{j}^{\left(r^{\prime }\right)}}\left(\begin{array}{c} N_{j}\\ l_{j}^{\left(r^{\prime }\right)} \end{array}\right)z_{j}^{N_{j}-\nu_{j}^{\left(r\right)}}\right]P\left(N,t\right)\\ \;\;\;\;\;\;\;\;-\overset{n_{s}}{\underset{j=1}{\prod }}\left[\Omega^{-l_{j}^{\left(r^{\prime }\right)}}\left(\begin{array}{c} N_{j}\\ l_{j}^{\left(r^{\prime }\right)} \end{array}\right)z_{j}^{N_{j}}\right]P\left(N,t\right)\} \end{array}$$

Finally, we can recast Equation (58) in a more simplified form:

(59) $$\partial_{t}G\left(z,t\right)=\overset{n_{r}}{\underset{r=1}{\sum }}\underset{N}{\sum }K_{r}^{\left(+\right)}\Omega \left\{\overset{n_{s}}{\underset{j=1}{\prod }}\Omega^{-s_{j}^{\left(r\right)}}\left(\overset{n_{s}}{\underset{j=1}{\prod }}\left(\begin{array}{c} N_{j}\\ s_{j}^{\left(r\right)} \end{array}\right)z_{j}^{N_{j}+\nu_{j}^{\left(r\right)}}-\overset{n_{s}}{\underset{j=1}{\prod }}\left(\begin{array}{c} N_{j}\\ s_{j}^{\left(r\right)} \end{array}\right)z_{j}^{N_{j}}\right)P\left(N,t\right)\right\}\;+\overset{n_{r}}{\underset{r=1}{\sum }}\underset{N}{\sum }K_{r}^{\left(-\right)}\Omega \left\{\overset{n_{s}}{\underset{j=1}{\prod }}\Omega^{-l_{j}^{\left(r\right)}}\left(\overset{n_{s}}{\underset{j=1}{\prod }}\left(\begin{array}{c} N_{j}\\ l_{j}^{\left(r\right)} \end{array}\right)z_{j}^{N_{j}-\nu_{j}^{\left(r\right)}}-\overset{n_{s}}{\underset{j=1}{\prod }}\left(\begin{array}{c} N_{j}\\ l_{j}^{\left(r\right)} \end{array}\right)z_{j}^{N_{j}}\right)P\left(N,t\right)\right\}$$

Equation (59) can be further simplified as follows, first we consider:

(60) $$\frac{\partial^{s_{j}^{\left(r\right)}}}{\partial z_{j}^{s_{j}^{\left(r\right)}}}z_{j}^{N_{j}}=N_{j}\left(N_{j}-1\right)\ldots \left(N_{j}-s_{j}^{\left(r\right)}+1\right)z_{j}^{N_{j}-s_{j}^{\left(r\right)}}$$

From Equation (60) the following identity is obtained:

(61) $$\left(\begin{array}{c} N_{j}\\ s_{j}^{\left(r\right)} \end{array}\right)z_{j}^{N_{j}}=\frac{z_{j}^{s_{j}^{\left(r\right)}}}{s_{j}^{\left(r\right)}!}\frac{\partial^{s_{j}^{\left(r\right)}}}{\partial z_{j}^{s_{j}^{\left(r\right)}}}z^{N_{j}}$$

The identity (60), when used in the Equation (59), yields the following result:

(62) $$\begin{array}{l} \partial_{t}G\left(z,t\right)=\overset{n_{r}}{\underset{r=1}{\sum }}K_{r}^{\left(+\right)}\Omega \left\{\overset{n_{s}}{\underset{j=1}{\prod }}\frac{\Omega^{-s_{j}^{\left(r\right)}}}{s_{j}^{\left(r\right)}!}\left(\overset{n_{s}}{\underset{j=1}{\prod }}z_{j}^{l_{j}^{\left(r\right)}}-\overset{n_{s}}{\underset{j=1}{\prod }}z_{j}^{s_{j}^{\left(r\right)}}\right)\overset{n_{s}}{\underset{j=1}{\prod }}\frac{\partial^{s_{j}^{\left(r\right)}}}{\partial z_{j}^{s_{j}^{\left(r\right)}}}G\left(z,t\right)\right\}\\ \;\;\;\;\;\;\;\;+\overset{n_{r}}{\underset{r^{\prime }=1}{\sum }}K_{r}^{\left(-\right)}\Omega \left\{\overset{n_{s}}{\underset{j=1}{\prod }}\frac{\Omega^{-l_{j}^{\left(r\right)}}}{l_{j}^{\left(r\right)}!}\left(\overset{n_{s}}{\underset{j=1}{\prod }}z_{j}^{s_{j}^{\left(r\right)}}-\overset{n_{s}}{\underset{j=1}{\prod }}z_{j}^{l_{j}^{\left(r\right)}}\right)\overset{n_{s}}{\underset{j=1}{\prod }}\frac{\partial^{l_{j}^{\left(r\right)}}}{\partial z_{j}^{l_{j}^{\left(r\right)}}}G\left(z,t\right)\right\} \end{array}$$

Finally, it is observed that the factor $\left(\prod_{j=1}^{n_{s}}z_{j}^{l_{j}^{\left(r\right)}}-\prod_{j=1}^{n_{s}}z_{j}^{s_{j}^{\left(r\right)}}\right)$ is common to both terms in Equation (62), so this equation can be further simplified to yield:

(63) $$\begin{array}{c} \partial_{t}G(z,t)=\overset{n_{r}}{\underset{r=1}{\sum }}[\overset{n_{s}}{\underset{j=1}{\prod }}z_{j}^{l_{j}^{\left(r\right)}}-\overset{n_{s}}{\underset{j=1}{\prod }}z_{j}^{s_{j}^{\left(r\right)}}](K_{r}^{\left(+\right)}\Omega \overset{n_{s}}{\underset{j=1}{\prod }}\frac{\Omega^{-s_{j}^{\left(r\right)}}}{s_{j}^{\left(r\right)}!}\frac{\partial^{s_{j}^{\left(r\right)}}}{\partial z_{j}^{s_{j}^{\left(r\right)}}}\\ -K_{r}^{\left(-\right)}\Omega \overset{n_{s}}{\underset{j=1}{\prod }}\frac{\Omega^{-l_{j}^{\left(r\right)}}}{l_{j}^{\left(r\right)}!}\frac{\partial^{l_{j}^{\left(r\right)}}}{\partial z_{j}^{l_{j}^{\left(r\right)}}})G\left(z,t\right) \end{array}$$

Equation (63) is the general partial differential equation (PDE) for the generating function of the chemical reaction networks. This equation is complex to solve in general due to its non-linear character but can be solved exactly for some simple cases.

To see that this equation is correct let us consider the chemical reaction $aA+bB\overset{K_{1}^{\left(+\right)}}{\to }cC+dD$, which is the example used by Smadbeck and Kaznessis [13]. For this example, the equation obeyed by the multiplicity generating function of this simple reaction because of Equation (63) is:

(64) $$\frac{\partial }{\partial t}G\left(z_{A},z_{B},t\right)=K_{1}^{\left(+\right)}\Omega \frac{1}{\Omega^{a}\Omega^{b}}\frac{1}{a!b!}\left(z_{C}^{c}z_{D}^{d}-z_{A}^{a}z_{B}^{b}\right)\frac{\partial^{a+b}}{\partial z_{A}^{a}\partial z_{B}^{b}}G\left(z_{A},z_{B},t\right)$$

Defining, $k_{1}=K_{1}^{\left(+\right)}\Omega \frac{1}{\Omega^{a}\Omega^{b}}$, it yields the same result that the one obtained by Smadbeck and Kaznessis.

### 3.2. The Method of the Expansion of the Chemical Master Equation in an Order Parameter 

As we have discussed previously, the fluctuations in the number of molecules of a given chemical compound $X_{j}$, are produced because of chemical reactions originate by collisions between the individual chemical compounds that are discrete in nature. Each chemical reaction produces one jump $\nu^{\left(r\right)}$, in the multidimensional vector space ${\mathbb{N}}^{n_{s}}$, the components of this jump $\nu_{j}^{\left(r\right)}$ are very smalls compared with the total number of molecules $N_{j}$ of each chemical species $X_{j}$. In addition, the macroscopic characteristics are due to the total population of each chemical compound. It is well known that for a population of $N_{j}$ molecules, the fluctuations are of the order of $\sqrt{N_{j}}$, and its effect on the macroscopic properties is of order NjNj~Nj−1/2. The election of the parameter to perform the expansion is usually the system volume Ω, because for large volumes the jumps are very small compared with the system population. There are different forms to perform this expansion, Van Kampen [8] proposed to split the dynamic of the molecular concentration in a deterministic part denoted by $\phi_{j}$ and a fluctuating component $\eta_{j}$, so that we can write down:

(65) $$N_{j}=\Omega \phi_{j}+\Omega^{1/2}\eta_{j},\;\mathrm{or}\;\frac{N_{j}}{\Omega }=\phi_{j}+\Omega^{-1/2}\eta_{j}$$

However, Samoilov and Arkin [48] proved that for some class of biochemical networks the mean behavior differs significantly from the rate equation of chemical classical kinetics (CCK). For this reason, especially for some class of biochemical networks involving bimolecular reactions, where the propensities according to the Equations (33) and (36) depend non-linearly on the populations, Andreychenko et al. [49] propose to perform an expansion around the true mean:

(66) $$\frac{N_{j}}{\Omega }=\langle \frac{N_{j}}{\Omega }\rangle +\Omega^{-1/2}{\bar{\eta }}_{j}$$

where from Equations (65) and (66) it is obtained that ${\bar{\eta }}_{j}$, is a random variable centered around zero and that quantifies the fluctuations around the true mean value.

To perform the systematic expansion of the Chemical Master Equation, first we write the CME (48) in the following form:

(67) $$\begin{array}{ll} \partial_{t}P\left(N,t\right)=\overset{n_{r}}{\underset{r=1}{\sum }}[ & \gamma_{r}\left(N-\nu^{\left(r\right)}\right)P\left(N-\nu^{\left(r\right)},t\right)-\gamma_{r}\left(N\right)P\left(N,t\right)]\\ & +\overset{n_{r}}{\underset{i=1}{\sum }}\left[\gamma_{n_{r}+i}\left(N+\nu^{\left(i\right)}\right)P\left(N+\nu^{\left(i\right)},t\right)-\gamma_{n_{r}+i}\left(N\right)P\left(N,t\right)\right] \end{array}$$

Next, we express the propensities for the direct reactions as follows:

(68) $$\gamma_{r}\left(N\right)=K_{r}^{\left(+\right)}\Omega \;\overset{n_{s}}{\underset{j=1}{\prod }}\Omega^{-s_{j}^{\left(r\right)}}\left(\begin{array}{c} N_{j}\\ s_{j}^{\left(r\right)} \end{array}\right).\;for\;r=1,2,\ldots ,n_{r}$$

and for the reverse reactions:

(69) $$\gamma_{n_{r}+i}\left(N\right)=K_{i}^{\left(-\right)}\Omega \;\overset{n_{s}}{\underset{j=1}{\prod }}\Omega^{-l_{j}^{\left(i\right)}}\left(\begin{array}{c} N_{j}\\ l_{j}^{\left(i\right)} \end{array}\right).\;for\;i=1,2,\ldots ,n_{r}$$

where $K_{r}^{\left(+\right)}=K_{r}$, and $K_{i}^{\left(-\right)}=K_{n_{r}+i}$. Therefore, the chemical master equation can be expressed because of Equations (67)–(69) in the form:

(70) $$\begin{array}{ll} \partial_{t}P\left(N,t\right)=\overset{n_{r}}{\underset{r=1}{\sum }}[ & K_{r}^{\left(+\right)}\Omega \;(\overset{n_{s}}{\underset{j=1}{\prod }}\Omega^{-s_{j}^{\left(r\right)}}\left(\begin{array}{c} N_{j}-\nu_{j}^{\left(r\right)}\\ s_{j}^{\left(r\right)} \end{array}\right)P\left(N-\nu^{\left(r\right)},t\right)\\ & -\overset{n_{s}}{\underset{j=1}{\prod }}\Omega^{-s_{j}^{\left(r\right)}}\left(\begin{array}{c} N_{j}\\ s_{j}^{\left(r\right)} \end{array}\right)P\left(N,t\right))]\\ & +\overset{n_{r}}{\underset{r=1}{\sum }}[K_{r}^{\left(-\right)}\Omega \;(\overset{n_{s}}{\underset{j=1}{\prod }}\Omega^{-l_{j}^{\left(r\right)}}\left(\begin{array}{c} N_{j}+\nu_{j}^{\left(r\right)}\\ l_{j}^{\left(r\right)} \end{array}\right)P\left(N+\nu^{\left(r\right)},t\right)\\ & -\overset{n_{s}}{\underset{j=1}{\prod }}\Omega^{-l_{j}^{\left(r\right)}}\left(\begin{array}{c} N_{j}\\ l_{j}^{\left(r\right)} \end{array}\right)P\left(N,t\right))] \end{array}$$

Next, one defines the translation operators in the multidimensional vector space ${\mathbb{N}}^{n_{s}}$, as follows:

(71) $$E^{\nu^{\left(r\right)}}P\left(N,t\right)=P\left(N+\nu^{\left(r\right)},t\right),\;\mathrm{and}\;E^{-\nu^{\left(r\right)}}P\left(N,t\right)=P\left(N-\nu^{\left(r\right)},t\right),$$

where:

(72) $$E^{\nu^{\left(r\right)}}=\prod_{j=1}^{n_{s}}E^{\nu_{j}^{\left(r\right)}}=\prod_{j=1}^{n_{s}}E^{l_{j}^{\left(r\right)}-s_{j}^{\left(r\right)}},\;\mathrm{and}\;E^{-\nu^{\left(r\right)}}=\prod_{j=1}^{n_{s}}E^{-\nu_{j}^{\left(r\right)}}=\prod_{j=1}^{n_{s}}E^{-\left(l_{j}^{\left(r\right)}-s_{j}^{\left(r\right)}\right)}$$

Using the operators defined in equations (71) and (72) in (70), the CME can be written down in the compact form:

(73) $$\begin{array}{ll} \partial_{t}P\left(N,t\right)=\sum_{r=1}^{n_{r}}[ & K_{r}^{\left(+\right)}\Omega \;\left(\overset{n_{s}}{\underset{j=1}{\prod }}\Omega^{-s_{j}^{\left(r\right)}}\left(E^{-\nu_{j}^{\left(r\right)}}-1\right)\left(\begin{array}{c} N_{j}\\ s_{j}^{\left(r\right)} \end{array}\right)P\left(N,t\right)\right)]\\ & +\overset{n_{r}}{\underset{r=1}{\sum }}\left[K_{r}^{\left(-\right)}\Omega \;\left(\overset{n_{s}}{\underset{j=1}{\prod }}\Omega^{-l_{j}^{\left(r\right)}}\left(E^{\nu_{j}^{\left(r\right)}}-1\right)\left(\begin{array}{c} N_{j}\\ l_{j}^{\left(r\right)} \end{array}\right)P\left(N,t\right)\right)\right] \end{array}$$

Next, we split the stochastic variable $N_{j}$ using Equation (65), in this approach the translation operators can be expanded in Taylor series [50]:

(74) $$E_{j}=\left(1+\frac{\partial }{\partial N_{j}}+\frac{1}{2}\frac{\partial^{2}}{\partial N_{j}^{2}}+..\right)=1+\Omega_{j}^{-1/2}\frac{\partial }{\partial \eta_{j}}+\Omega^{-1}\frac{\partial^{2}}{\partial \eta_{j}^{2}}+..$$

(75) $$E_{j}^{-1}=\left(1-\frac{\partial }{\partial N_{j}}+\frac{1}{2}\frac{\partial^{2}}{\partial N_{j}^{2}}+..\right)=1-\Omega_{j}^{-1/2}\frac{\partial }{\partial \eta_{j}}+\Omega^{-1}\frac{\partial^{2}}{\partial \eta_{j}^{2}}+..$$

Therefore, assuming that $\nu_{j}^{\left(r\right)}>0$, the operators $E_{j}^{\nu_{j}^{\left(r\right)}}$ and $E_{j}^{-\nu_{j}^{\left(r\right)}},$ on account that the $\nu_{j}^{\left(r\right)}$ are integer numbers and retaining only the first three terms of the $E_{j}$ expansion can be written as follows:

(76) $$E_{j}^{\nu_{j}^{\left(r\right)}}={\left(1+\Omega^{-1/2}\frac{\partial }{\partial \eta_{j}}+\Omega^{-1}\frac{\partial^{2}}{\partial \eta_{j}^{2}}\right)}^{\nu_{j}^{\left(r\right)}}=\underset{\begin{array}{c} i,q,k\\ i+q+k=\nu_{j}^{\left(r\right)} \end{array}}{\sum }\left(\begin{array}{c} \nu_{j}^{\left(r\right)}\\ i,q,k \end{array}\right){\left(\Omega^{-1/2}\frac{\partial }{\partial \eta_{j}}\right)}^{q}{\left(\Omega^{-1}\frac{\partial^{2}}{\partial \eta_{j}^{2}}\right)}^{k}$$

(77) $$E_{j}^{-\nu_{j}^{\left(r\right)}}={\left(1-\Omega^{-1/2}\frac{\partial }{\partial \eta_{j}}+\Omega^{-1}\frac{\partial^{2}}{\partial \eta_{j}^{2}}\right)}^{\nu_{j}^{\left(r\right)}}=\sum_{\begin{array}{c} i,q,k\\ i+q+k=\nu_{j}^{\left(r\right)} \end{array}}\left(\begin{array}{c} \nu_{j}^{\left(r\right)}\\ i,q,k \end{array}\right){\left(-\Omega^{-1/2}\frac{\partial }{\partial \eta_{j}}\right)}^{q}{\left(\Omega^{-1}\frac{\partial^{2}}{\partial \eta_{j}^{2}}\right)}^{k},$$

where the multinomial coefficients [51] are defined in the standard way:

(78) $$\left(\begin{array}{c} \nu_{j}^{\left(r\right)}\\ i,q,k \end{array}\right)=\frac{\nu_{j}^{\left(r\right)}!}{i!\;q!\;k!}$$

Also, it is observed that the partial derivative with respect to *t*, is computed at N constant for all its components, therefore we can write:

(79) $$\partial_{t}P\left(N,t\right)=\partial_{t}\Pi \left(\eta ,t\right)+\sum_{j=1}^{n_{s}}\frac{\partial \Pi \left(\eta ,t\right)}{\partial \eta_{j}}\frac{d\eta_{j}}{dt}=\partial_{t}\Pi \left(\eta ,t\right)-\Omega^{1/2}\sum_{j=1}^{n_{s}}\frac{\partial \Pi \left(\eta ,t\right)}{\partial \eta_{j}}\frac{d\phi_{j}}{dt},$$

where to compute the time derivative $\frac{d\eta_{j}}{dt}$, we have considered that at $N_{j}=cte$, and because of $\frac{N_{j}}{\Omega }=\phi_{j}+\Omega^{-1/2}\eta_{j}$, then it is deduced that $\frac{d\eta_{j}}{dt}=-\Omega^{1/2}\frac{d\phi_{j}}{dt}$. Therefore, the final form for the probability of the fluctuations is obtained because of Equations (76), (77) and (79) in Equation (73), which yields the following result:

(80) $$\begin{array}{l} \partial_{t}\Pi \left(\eta ,t\right)-\Omega^{1/2}\overset{n_{s}}{\underset{j=1}{\sum }}\frac{\partial \Pi \left(\eta ,t\right)}{\partial \eta_{j}}\frac{d\phi_{j}}{dt}\\ =\sum_{r=1}^{n_{r}}\left[K_{r}^{\left(+\right)}\Omega \overset{n_{s}}{\underset{j=1}{\prod }}\Omega^{-s_{j}^{\left(r\right)}}\;\left(\underset{\begin{array}{c} i,q,k\\ i+q+k=\nu_{j}^{\left(r\right)}\\ i\neq \nu_{j}^{\left(r\right)} \end{array}}{\sum }\left(\begin{array}{c} \nu_{j}^{\left(r\right)}\\ i,q,k \end{array}\right){\left(-\Omega^{-1/2}\frac{\partial }{\partial \eta_{j}}\right)}^{q}{\left(\Omega^{-1}\frac{\partial^{2}}{\partial \eta_{j}^{2}}\right)}^{k}\left(\begin{array}{c} \Omega \phi_{j}+\Omega^{1/2}\eta_{j}\\ s_{j}^{\left(r\right)} \end{array}\right)\Pi \left(\eta ,t\right)\right)\right]\\ +\sum_{r=1}^{n_{r}}\left[K_{r}^{\left(-\right)}\Omega \;\overset{n_{s}}{\underset{j=1}{\prod }}\Omega^{-l_{j}^{\left(r\right)}}\left(\underset{\begin{array}{c} i,q,k\\ i+q+k=\nu_{j}^{\left(r\right)}\\ i\neq \nu_{j}^{\left(r\right)} \end{array}}{\sum }\left(\begin{array}{c} \nu_{j}^{\left(r\right)}\\ i,q,k \end{array}\right){\left(\Omega^{-1/2}\frac{\partial }{\partial \eta_{j}}\right)}^{q}{\left(\Omega^{-1}\frac{\partial^{2}}{\partial \eta_{j}^{2}}\right)}^{k}\left(\begin{array}{c} \Omega \phi_{j}+\Omega^{1/2}\eta_{j}\\ l_{j}^{\left(r\right)} \end{array}\right)\Pi \left(\eta ,t\right)\right)\right] \end{array}$$

If $\nu_{j}^{\left(r\right)}<0$, then $-\nu_{j}^{\left(r\right)}>0$, and $E_{j}^{\nu_{j}^{\left(r\right)}}={\left(E_{j}^{-1}\right)}^{-\nu_{j}^{\left(r\right)}}$, therefore for this case from Equation (73) it is deduced:

(81) $$\begin{array}{l} \partial_{t}\Pi \left(\eta ,t\right)-\Omega^{1/2}\overset{n_{s}}{\underset{j=1}{\sum }}\frac{\partial \Pi \left(\eta ,t\right)}{\partial \eta_{j}}\frac{d\phi_{j}}{dt}\\ =\sum_{r=1}^{n_{r}}\left[K_{r}^{\left(+\right)}\Omega \overset{n_{s}}{\underset{j=1}{\prod }}\Omega^{-s_{j}^{\left(r\right)}}\;\left(\underset{\begin{array}{c} i,q,k\\ i+q+k=-\nu_{j}^{\left(r\right)}\\ i\neq -\nu_{j}^{\left(r\right)} \end{array}}{\sum }\left(\begin{array}{c} -\nu_{j}^{\left(r\right)}\\ i,q,k \end{array}\right){\left(\Omega^{-1/2}\frac{\partial }{\partial \eta_{j}}\right)}^{q}{\left(\Omega^{-1}\frac{\partial^{2}}{\partial \eta_{j}^{2}}\right)}^{k}\left(\begin{array}{c} \Omega \phi_{j}+\Omega^{1/2}\eta_{j}\\ s_{j}^{\left(r\right)} \end{array}\right)\Pi \left(\eta ,t\right)\right)\right]\\ +\sum_{r=1}^{n_{r}}\left[K_{r}^{\left(-\right)}\Omega \;\overset{n_{s}}{\underset{j=1}{\prod }}\Omega^{-l_{j}^{\left(r\right)}}\left(\underset{\begin{array}{c} i,q,k\\ i+q+k=-\nu_{j}^{\left(r\right)}\\ i\neq -\nu_{j}^{\left(r\right)} \end{array}}{\sum }\left(\begin{array}{c} -\nu_{j}^{\left(r\right)}\\ i,q,k \end{array}\right){\left(-\Omega^{-1/2}\frac{\partial }{\partial \eta_{j}}\right)}^{q}{\left(\Omega^{-1}\frac{\partial^{2}}{\partial \eta_{j}^{2}}\right)}^{k}\left(\begin{array}{c} \Omega \phi_{j}+\Omega^{1/2}\eta_{j}\\ l_{j}^{\left(r\right)} \end{array}\right)\Pi \left(\eta ,t\right)\right)\right] \end{array}$$

Obviously, the expansion in Equation (80) can be performed at higher orders and in this case higher order multinomial coefficients will appear in (80) and (81).

If we use an expansion around the true mean as in Equation (66), then the partial derivative with respect to t, is computed at N constant for all its components, therefore we can write:

(82) $$\partial_{t}P\left(N,t\right)=\partial_{t}\bar{\Pi }\left(\bar{\eta },t\right)+\sum_{j=1}^{n_{s}}\frac{\partial \bar{\Pi }\left(\bar{\eta },t\right)}{\partial {\bar{\eta }}_{j}}\frac{d{\bar{\eta }}_{j}}{dt}=\partial_{t}\Pi \left(\bar{\eta },t\right)-\Omega^{1/2}\sum_{j=1}^{n_{s}}\frac{\partial \bar{\Pi }\left(\bar{\eta },t\right)}{\partial {\bar{\eta }}_{j}}\frac{d}{dt}\frac{\langle N_{j}\rangle }{\Omega },$$

Also, in this approach the translation operators can be expanded in Taylor series:

(83) $$E_{j}=\left(1+\frac{\partial }{\partial N_{j}}+\frac{1}{2}\frac{\partial^{2}}{\partial N_{j}^{2}}+..\right)=1+\Omega_{j}^{-1/2}\frac{\partial }{\partial {\bar{\eta }}_{j}}+\Omega^{-1}\frac{\partial^{2}}{\partial {\bar{\eta }}_{j}^{2}}+\ldots$$

Then, if $\nu_{j}^{\left(r\right)}>0$, it is easy to prove that the probability $\bar{\Pi }\left(\bar{\eta },t\right)$ of the fluctuations around the mean obeys the following equation:

(84) $$\begin{array}{l} \partial_{t}\bar{\Pi }\left(\bar{\eta },t\right)-\Omega^{1/2}\overset{n_{s}}{\underset{j=1}{\sum }}\frac{\partial \bar{\Pi }\left(\bar{\eta },t\right)}{\partial \eta_{j}}\frac{d}{dt}\;\frac{\langle N_{j}\rangle }{\Omega }\\ =\sum_{r=1}^{n_{r}}\left[K_{r}^{\left(+\right)}\Omega \overset{n_{s}}{\underset{j=1}{\prod }}\Omega^{-s_{j}^{\left(r\right)}}\left(\underset{\begin{array}{c} i,q,k\\ i+q+k=\nu_{j}^{\left(r\right)}\\ i\neq \nu_{j}^{\left(r\right)} \end{array}}{\sum }\left(\begin{array}{c} \nu_{j}^{\left(r\right)}\\ i,q,k \end{array}\right){\left(-\Omega^{-1/2}\frac{\partial }{\partial {\bar{\eta }}_{j}}\right)}^{q}{\left(\Omega^{-1}\frac{\partial^{2}}{\partial {\bar{\eta }}_{j}^{2}}\right)}^{k}\left(\begin{array}{c} \Omega \left(\frac{\langle N_{j}\rangle }{\Omega }\right)+\Omega^{1/2}{\bar{\eta }}_{j}\\ s_{j}^{\left(r\right)} \end{array}\right)\bar{\Pi }\left(\bar{\eta },t\right)\right)\right]\\ +\sum_{r=1}^{n_{r}}\left[K_{r}^{\left(-\right)}\Omega \;\overset{n_{s}}{\underset{j=1}{\prod }}\Omega^{-l_{j}^{\left(r\right)}}\left(\underset{\begin{array}{c} i,q,k\\ i+q+k=\nu_{j}^{\left(r\right)}\\ i\neq \nu_{j}^{\left(r\right)} \end{array}}{\sum }\left(\begin{array}{c} \nu_{j}^{\left(r\right)}\\ i,q,k \end{array}\right){\left(\Omega^{-1/2}\frac{\partial }{\partial {\bar{\eta }}_{j}}\right)}^{q}{\left(\Omega^{-1}\frac{\partial^{2}}{\partial {\bar{\eta }}_{j}^{2}}\right)}^{k}\left(\begin{array}{c} \Omega \left(\frac{\langle N_{j}\rangle }{\Omega }\right)+\Omega^{1/2}{\bar{\eta }}_{j}\\ l_{j}^{\left(r\right)} \end{array}\right)\bar{\Pi }\left(\bar{\eta },t\right)\right)\right] \end{array}$$

A similar equation to (84) can be also deduced for the case $\nu_{j}^{\left(r\right)}<0$.

## 4. Methods to Obtain the Probability Distribution from the Moments

The CME has an analytical solution in a few cases. The handbook of stochastic methods by Gardiner provides several examples of exactly solvable models of the CME [40]. More examples can be found in references [52,53]. In the rest of cases the solution of the CME can be found by Monte-Carlo methods, by the finite state projection method [12], or reconstructing the PDF from the moments as it is shown below.

### Reconstructing the Probability Distribution from the Moments for One Component Case

For this case, we assume for simplicity a single component, and that at some time instant t, the moments $\mu_{k}\;k=1,2,\ldots ,NM,$ of the distribution are known, where *NM* is the number of moments. If P(N,t), denotes the probability to have *N* molecules at time *t*, then we can write the following set of conditions:

(85) $$\mu_{k}\left(t\right)=\sum_{N}N^{k}P\left(N,t\right),\;\;\;k=1,2,\ldots ,\;NM,$$

In addition, we have the normalization condition for the probability, so that

(86) $$\mu_{0}\left(t\right)=\underset{N}{\sum }P\left(N,t\right)=1$$

Then following the maximum entropy principle [29,30,49], the probability distribution function P(N,t), which must be chosen, is the one that maximizes the Shannon information entropy and at the same time satisfies the restrictions (85) imposed by the moments and the probability normalization condition (86). This problem is a typical optimization problem with constraints that can be solved using the Lagrange multipliers method by building the functional:

(87) $$F\left(P,\lambda \right)=-\underset{N}{\sum }P\left(N,t\right)logP\left(N,t\right)+\overset{NM}{\underset{k=0}{\sum }}\lambda_{k}\left(\underset{N}{\sum }N^{k}P\left(N,t\right)-\mu_{k}\left(t\right)\right)$$

This optimization problem leads to the following solution, known as the maximum-entropy PDF:

(88) $$P\left(N,t\right)=\exp(-1+\lambda_{0}+\overset{NM}{\underset{k=1}{\sum }}\lambda_{k\;}N^{k})$$

The Lagrange multipliers are obtained from the restriction conditions (85) and (86). Applying the normalization condition (86), to Equation (88), it is obtained:

(89) $$Z={\left(\exp\left(-1+\lambda_{0}\right)\right)}^{-1}=\underset{N}{\sum }\exp(\overset{NM}{\underset{k=0}{\sum }}\lambda_{k\;}N^{k})$$

so, the probability distribution that maximizes the information entropy is given by the expression:

(90) $$P\left(N,t\right)=Z^{-1}\exp(\overset{NM}{\underset{k=1}{\sum }}\lambda_{k\;}N^{k}),$$

where the unknown *Z* depends on the Lagrange multipliers. There exist different algorithms to obtain the Lagrange multipliers from the moments as explained in Andreychenko et al. and Woodbury [49,54]. 

The previous deduction can be generalized to the case of the moment evolution with time, in this case the time can be considered as a parameter, and therefore we can use variational calculus as in Caticha and Preuss [55]. Because we must maximize the entropy at each time instant then we look for the time function P(N,t), which makes maximum the information entropy along time and at the same time satisfy the restrictions imposed by the moments, notice that for this case the Lagrange multipliers should depend on time, so we write the functional in the form:

(91) $$F\left(P,\lambda \right)=\int_{t_{0}}^{t_{f}}-\overset{\infty }{\underset{N=0}{\sum }}P\left(N,t\right)logP\left(N,t\right)dt+\;\overset{NM}{\underset{j=0}{\sum }}\overset{t_{f}}{\underset{t_{0}}{\int }}dt\lambda_{j}\left(t\right)\left(\underset{N}{\sum }N^{j}P\left(N,t\right)-\mu_{j}\left(t\right)\right)$$

Then, we perform the variations of the PDF and the Lagrange multipliers that are now function of the parameter i.e., P(N,t)+ϵδP(N,t), and $\lambda_{j}\left(t\right)+\epsilon \delta \lambda_{j}\left(t\right)$, and we look for the probability distribution that makes δF=0. Where the first variation is computed using standard rules of variational calculus as follows:

(92) $$\delta F={\left[\frac{\partial F(P+\epsilon \delta P,\;\;\;\lambda +\epsilon \delta \lambda }{\partial \epsilon }\right]}_{\epsilon =0}=0$$

Performing the operation (92) in the functional (91) one arrives to the following equation:

(93) $$\begin{array}{ll} \delta F\left(P,\lambda \right)=\int_{t_{0}}^{t_{f}}dt & \overset{\infty }{\underset{N=0}{\sum }}\left\{-logP\left(N,t\right)+1+\overset{NM}{\underset{j=0}{\sum }}\lambda_{j}\left(t\right)N^{j}\right\}\delta P\left(N,t\right)dt\\ & +\overset{NM}{\underset{j=0}{\sum }}\overset{t_{f}}{\underset{t_{0}}{\int }}dt\delta \lambda_{j}\left(t\right)\left(\underset{N}{\sum }N^{j}P\left(N,t\right)-\mu_{j}\left(t\right)\right)=0 \end{array}$$

Due to the arbitrary character of the functions δP(N,t) and $\delta \lambda_{j}\left(t\right),\;j=0,1,\ldots ,NM$, one arrives to:

(94) $$P\left(N,t\right)=\exp(-1+\lambda_{0}\left(t\right)+\overset{NM}{\underset{k=1}{\sum }}\lambda_{k\;}\left(t\right)N^{k})$$

where now the Lagrange multipliers are functions of t, also it is obtained the set of restrictions (85), and (86), with the probability P(N,t), given by (94).

We can use another moment basis, which is known as the factorial moment basis; in this case for one component we define the following set of population functions:

(95) $$g_{q}^{\left(f\right)}\left(N\right)=N\left(N-1\right)\ldots \left(N-q+1\right)$$

Then, the factorial moments of order q denoted by ${\left\{N^{q}\right\}}_{f}$ are defined by the following set of equations:

(96) $${\left\{N^{q}\right\}}_{f}=\langle g_{q}^{\left(f\right)}\left(N\right)\rangle =\langle N\left(N-1\right)\ldots \left(N-q+1\right)\rangle$$

If one knows the factorial moments up to a given order *M*, at time *t*, then using the MEP one can obtain the probability distribution that maximizes the information entropy and at the same time satisfy the restrictions imposed by the moments. In this case it is obtained the following result:

(97) $$P\left(N,t\right)=\exp(-1+\lambda_{0}^{\prime }\left(t\right)+\overset{M}{\underset{q=1}{\sum }}\lambda_{q}^{\prime }\left(t\right)g_{q}^{\left(f\right)}\left(N\right))$$

The Lagrange multipliers $\lambda_{q}^{\prime }$ are obtained solving the system of equations:

(98) $${\left\{N^{q}\right\}}_{f}=\underset{N}{\sum }g_{q}^{\left(f\right)}\left(N\right)P\left(N,t\right),q=1,2,\ldots ,M$$

(99) $$\underset{N}{\sum }P\left(N,t\right)=1$$

## 5. Results of Applications to Chemical Reactions Networks

In this section we apply the methods explained previously to some specific chemical reaction networks. Beginning with very well-known cases and finishing with complex ones. Also, we study in this section the consistency of the results obtained by the expansion of the CME in an order parameter, by comparison with the results obtained using the PGF method. Finally, because of its importance in practical applications we have included a section devoted to the different methods to solve the closure problem.

### 5.1. Case of One Single Component in an Open System

One of the simplest chemical reaction networks, which contain non-linear terms, is the following one:

(100) $$A\overset{K_{1}^{\left(+\right)}}{\to }X$$

(101) $$X+X\overset{K_{2}^{\left(+\right)}}{\to }B$$

where it is assumed that the reaction takes place in a well stirred tank, being the compound *A* supplied continuously, so the amount of *A* remains constant and the chemical compound *B* is also removed continuously so *B* is also assumed constant. In this example we denote the reaction (100) as reaction 1 and the reaction (101) as the reaction (2). For these reactions $\sum_{j}^{n_{s}}s_{j}^{\left(r\right)}\to \sum_{j}^{n_{s}}l_{j}^{\left(r\right)}$ we have that the stochiometric coefficients and the jumps are:

(102) $$r=1,\;s_{x}^{\left(1\right)}=0,\;l_{x}^{\left(1\right)}=1,\;\;\nu_{x}^{\left(1\right)}=1,\;s_{A}^{\left(1\right)}=1,\;l_{A}^{\left(1\right)}=0,\;\nu_{A}^{\left(1\right)}=-1$$

(103) $$r=2,\;s_{x}^{\left(2\right)}=2,\;l_{x}^{\left(2\right)}=0,\;\;\nu_{x}^{\left(2\right)}=-2$$

Then Equation (80) for the network formed by the direct chemical reactions (101) and (102) can be written down as follows:

(104) $$\begin{array}{ll} \partial_{t}\Pi \left(\eta_{x},t\right)-\Omega^{\frac{1}{2}} & \frac{\partial \Pi \left(\eta_{x},t\right)}{\partial \eta_{x}}\frac{d\phi_{x}}{dt}\\ & =\Omega K_{1}^{\left(+\right)}\Omega^{-1}\left(\begin{array}{c} \Omega \phi_{A}\\ 1 \end{array}\right)\left[-\Omega^{-\frac{1}{2}}\frac{\partial \Pi \left(\eta_{x},t\right)}{\partial \eta_{x}}+\frac{1}{2}\Omega^{-1}\frac{\partial^{2}\Pi \left(\eta_{x},t\right)}{\partial \eta_{x}^{2}}\right]\\ & +\Omega K_{2}^{\left(+\right)}\Omega^{-2}[\left(\begin{array}{c} 2\\ 1,1,0 \end{array}\right)\left(\Omega^{-1/2}\frac{\partial }{\partial \eta_{x}}\right)+\left(\begin{array}{c} 2\\ 1,0,1 \end{array}\right)\left(\frac{1}{2}\Omega^{-1}\frac{\partial^{2}}{\partial \eta_{x}^{2}}\right)\\ & +\left(\begin{array}{c} 2\\ 0,1,1 \end{array}\right)\left(\Omega^{-1/2}\frac{\partial }{\partial \eta_{x}}\right)\left(\frac{1}{2}\Omega^{-1}\frac{\partial^{2}}{\partial \eta_{x}^{2}}\right)+\left(\begin{array}{c} 2\\ 0,2,0 \end{array}\right){\left(\Omega^{-1/2}\frac{\partial }{\partial \eta_{x}}\right)}^{2}\\ & +\left(\begin{array}{c} 2\\ 0,0,2 \end{array}\right){\left(\frac{1}{2}\Omega^{-1}\frac{\partial^{2}}{\partial \eta_{x}^{2}}\right)}^{2}]\left(\begin{array}{c} \Omega \phi_{x}+\Omega^{1/2}\eta_{x}\\ 2 \end{array}\right)\Pi \left(\eta_{x},t\right) \end{array}$$

At order $\Omega^{1/2}$, i.e., collecting all the terms proportional to $\Omega^{1/2}$, and which are the biggest ones, we have the following equation:

(105) $$\Omega^{1/2}\left\{\frac{d\phi_{x}}{dt}-K_{1}^{\left(+\right)}\phi_{A}+K_{2}^{\left(+\right)}\phi_{x}^{2}\right\}\frac{\partial \Pi \left(\eta_{x},t\right)}{\partial \eta_{x}}=0$$

From Equation (105) it is deduced the macroscopic equation of chemical kinetic for the macroscopic concentration $\phi_{x}$ for the open systems chemical network (100) and (101):

(106) $$\frac{d\phi_{x}}{dt}=K_{1}^{\left(+\right)}\phi_{A}-K_{2}^{\left(+\right)}\phi_{x}^{2}$$

We observe that in Van-Kampen book [8] the coefficient of the term $\phi_{x}^{2}$ is 2. The reason of this is that $K_{2}^{\left(+\right)}$ = 2K, because the propensities were defined in Section 2 per pair of interacting particles, and the number of couples when the particles are identical is divided by 2, because it is given by the combinatorial number $\left(\begin{array}{c} N\\ 2 \end{array}\right)$.

The next step is to collect all the terms of order $\Omega^{0}$, which after some simple algebra yields:

(107) $$\begin{array}{l} \Omega^{0}\left\{-\partial_{t}\Pi \left(\eta_{x},t\right)+\frac{1}{2}K_{1}^{\left(+\right)}\phi_{A}\frac{\partial^{2}}{\partial \eta_{x}^{2}}\Pi \left(\eta_{x},t\right)+K_{2}^{\left(+\right)}\phi_{x}^{2}\frac{\partial^{2}}{\partial \eta_{x}^{2}}\Pi \left(\eta_{x},t\right)+2K_{2}^{\left(+\right)}\phi_{x}\frac{\partial }{\partial \eta_{x}}\left(\eta_{x}\Pi \left(\eta_{x},t\right)\right)\right\}\\ \;\;\;\;\;\;=0 \end{array}$$

From, Equation (107) at this order $\Omega^{0}$ it is obtained the following equation for the probability $\Pi \left(\eta_{x},t\right)$:

(108) $$\partial_{t}\Pi \left(\eta_{x},t\right)=2K_{2}^{\left(+\right)}\phi_{x}\left(t\right)\frac{\partial }{\partial \eta_{x}}\left(\eta_{x}\Pi \left(\eta_{x},t\right)\right)+\left(\frac{1}{2}K_{1}^{\left(+\right)}\phi_{A}+K_{2}^{\left(+\right)}\phi_{x}^{2}\right)\frac{\partial^{2}}{\partial \eta_{x}^{2}}\Pi \left(\eta_{x},t\right)$$

According to Risken’s book [56], Equation (108) is a linear Fokker-Plank partial differential equation with time dependent coefficients through $\phi_{x}\left(t\right)$. This equation is the same one obtained by Van Kampen [8] at the same order. The solution of Equation (108) leads to a Gaussian distribution as shown by Van Kampen [8]. Therefore, we only need to obtain the first two moments of the distribution to know its PDF:

(109) $$\langle \eta_{x}\rangle =\int_{D}\eta_{x}\Pi \left(\eta_{x},t\right)d\eta_{x},\;\langle \eta_{x}^{2}\rangle =\int_{D}\eta_{x}^{2}\Pi \left(\eta_{x},t\right)d\eta_{x},\;\mathrm{and}\;\sigma^{2}\left(\eta_{x}\right)=\langle \eta_{x}^{2}\rangle -\langle \eta_{x}\rangle {}^{2}$$

where D is the distribution support. Obviously, if we go to higher orders of the expansion, the equation that governs the evolution of the probability is not Equation (108) and will contain additional terms and higher derivatives. 

The next step is to obtain the equations that govern the evolution equation for the first and second moments, in this case from Equation (108), because of (109) it is obtained:

(110) $$\partial_{t}\langle \eta_{x}\rangle =-2K_{2}^{\left(+\right)}\phi_{x}\langle \eta_{x}\rangle$$

(111) $$\partial_{t}\langle \eta_{x}^{2}\rangle =-4K_{2\;}^{\left(+\right)}\phi_{x}\left(t\right)\langle \eta_{x}^{2}\rangle +\left(K_{1}^{\left(+\right)}\phi_{A}+2K_{2}^{\left(+\right)}\phi_{x}^{2}\right)$$

Let us study now the reaction network formed by (100) and (101) by the method of the moment generating function, in this case we assume also that the population of *A* is constant, and that *B* is also removed continuously to maintain *B* also constant. For this simple case the generating function evolution equation of this reaction network is given because of Equations (63), (102) and (103) by:

(112) $$\partial_{t}G\left(z_{x},t\right)=\left(z_{x}-1\right)K_{1}^{\left(+\right)}N_{A}G\left(z_{x},t\right)+\left(1-z_{x}^{2}\right)\frac{K_{2}^{\left(+\right)}}{2}\Omega^{-1}\frac{\partial^{2}}{\partial z_{x}^{2}}G\left(z_{x},t\right)$$

Application to Equation (112) of the operator ${\left[\frac{\partial }{\partial z_{x}}\right]}_{z_{x}=1}$ yields for the first moment $\langle N_{x}\rangle$ the following result:

(113) $$\frac{d}{dt}\langle N_{x}\rangle =K_{1}^{\left(+\right)}N_{A}-K_{2}^{\left(+\right)}\Omega^{-1}\langle N_{x}\left(N_{x}-1\right)\rangle$$

Defining the variation $\delta N_{x}$ of the number of molecules of the chemical compound X respect to the mean value as $\delta N_{x}=N_{x}-\langle N_{X}\rangle$, it is obtained:

(114) $$\langle \delta N_{x}^{2}\rangle =\langle N_{x}^{2}\rangle -\langle N_{x}\rangle {}^{2}=\sigma^{2}\left(N_{x}\right)$$

From (113) and (114), it is deduced that:

(115) $$\frac{d}{dt}\langle N_{x}\rangle =K_{1}^{\left(+\right)}N_{A}-K_{2}^{\left(+\right)}\Omega^{-1}\langle N_{x}\rangle {}^{2}+K_{2}^{\left(+\right)}\Omega^{-1}\left(\sigma^{2}\left(N_{x}\right)-\langle N_{x}\rangle \right)$$

Defining an average concentration of *X* as ${\bar{\phi }}_{x}$ = $\langle N_{x}\rangle /\Omega$, it is obtained after dividing Equation (115) by the system volume Ω:

(116) $$\frac{d}{dt}{\bar{\phi }}_{x}=K_{1}^{\left(+\right)}\phi_{A}-K_{2}^{\left(+\right)}{\bar{\phi }}_{x}{}^{2}+K_{2}^{\left(+\right)}\Omega^{-2}\left(\sigma^{2}-\langle N_{x}\rangle \right)$$

Only if the probability distribution follows a Poisson PDF, we have that $\sigma^{2}=\langle N_{x}\rangle$, and in this case Equation (116) simplifies to:

(117) $$\frac{d}{dt}{\bar{\phi }}_{x}=K_{1}^{\left(+\right)}\phi_{A}-K_{2}^{\left(+\right)}{\bar{\phi }}_{x}{}^{2}\;\mathrm{only}\;\mathrm{if}\;P\left(N_{x},t\right)\;\mathrm{is}\;a\;\mathrm{Poisson}\;\mathrm{PDF}$$

But in general, this is not true, so that in the equation for the mean average appear terms that depend on the fluctuations. In addition, as we will see below, the evolution equation for any order moment depends on higher order moments.

Application of the operator ${\left[\frac{\partial^{2}}{\partial z_{x}^{2}}\right]}_{z_{x}=1}$ to the equation (112) of the moment generating function yields after some calculus:

(118) $$\frac{d}{dt}\langle N_{x}(N_{x}-1)\rangle =2\;K_{1}^{\left(+\right)}N_{A}\langle N_{x}\rangle -K_{2}^{\left(+\right)}\Omega^{-1}\langle N_{x}(N_{x}-1)\rangle -2K_{2}^{\left(+\right)}\Omega^{-1}\langle N_{x}\left(N_{x}-1\right)(N_{x}-2)\rangle$$

Equation (118) tell us that the evolution equation for the second factorial moments depends on the first, second and third factorial moment. Let us check now the consistency of this result, to do that let us assume that the PDF follows a Poisson distribution. Then as it is well known the factorial moments ${\left\{N_{x}^{q}\right\}}_{f}$ of a Poisson PDF verify the following conditions, see the book of Gardiner [40]:

(119) $${\left\{N_{x}^{q}\right\}}_{f}=\langle N_{x}(N_{x}-1)\ldots (N_{x}-q+1)\rangle ={\langle N_{x}\rangle }^{q}\;\mathrm{for}\;q=1,2,\ldots$$

Then, because of (119), the right-hand side of Equation (118) yields if the PDF is a Poisson one:

(120) $$\frac{d}{dt}{\left\{N_{x}^{2}\right\}}_{f}=2\;K_{1}^{\left(+\right)}N_{A}\langle N_{x}\rangle -K_{2}^{\left(+\right)}\Omega^{-1}\langle N_{x}\rangle {}^{2}-2K_{2}^{\left(+\right)}\Omega^{-1}\langle N_{x}\rangle {}^{3}$$

Then the left-hand side of Equation (118), yields:

(121) $$\frac{d}{dt}{\left\{N_{x}^{2}\right\}}_{f}=\frac{d}{dt}{\langle N_{x}\rangle }^{2}=2\langle N_{x}\rangle \frac{d}{dt}\langle N_{x}\rangle =2K_{1}^{\left(+\right)}N_{A}\langle N_{x}\rangle -2K_{2}^{\left(+\right)}\Omega^{-1}{\langle N_{x}\rangle }^{3}$$

Apparently, the right-hand side of Equation (120) contains the term $-K_{2}^{\left(+\right)}\Omega^{-1}\langle N_{x}\rangle {}^{2}$, that does not show up in Equation (121). But dividing both Equations (120) and (121) by $\Omega^{2}$, and expressing the magnitudes in terms of average macroscopic concentrations it is obtained from (120) and (121) the following results:

(122) $$\frac{d}{dt}\frac{{\left\{N_{x}^{2}\right\}}_{f}}{\Omega^{2}}=2\;K_{1}^{\left(+\right)}\phi_{A}{\bar{\phi }}_{x}-K_{2}^{\left(+\right)}\Omega^{-1}{\bar{\phi }}_{x}{}^{2}+2K_{2}^{\left(+\right)}{\bar{\phi }}_{x}{}^{3}$$

(123) $$\frac{d}{dt}\frac{{\left\{N_{x}^{2}\right\}}_{f}}{\Omega^{2}}=2\;K_{1}^{\left(+\right)}\phi_{A}{\bar{\phi }}_{x}+2K_{2}^{\left(+\right)}{\bar{\phi }}_{x}{}^{3}$$

So, it is deduced that the difference between both equations is the term $-K_{2}^{\left(+\right)}\Omega^{-1}{\bar{\phi }}_{x}{}^{2}$. It is observed that this term is much smaller than the other two terms because is divided by the order parameter Ω that is the system volume, that is generally large, and makes this term very small compared with the other two terms so the apparently contradiction for poissonian systems is solved.

### 5.2. Examples of Two Component Chemical Reaction Networks

One simple example of two component chemical reaction networks in a closed system with non-linear effect is the one studied by Smadbeck and Kaznessis [13] and consist in the reversible dimerization of two monomers. In this case we have only one reaction and its inverse, i.e., the reaction of two monomers *A* to form a dimer *B* and at the same time the decomposition of this dimer B in the two original monomers A. Each reaction has its own constant, so we write:

(124) $$A+A\begin{array}{c} \overset{K_{1}^{\left(+\right)}}{\to }\\ \underset{K_{1}^{\left(-\right)}}{\leftarrow } \end{array}B$$

For this case the stoichiometric coefficients and the jumps are:

(125) $$s_{A}=2,\;\;l_{A}=0,\;s_{B}=0,\;\;l_{B}=1,\;\nu_{A}=-2,\;\nu_{B}=1$$

The next step is to apply Equation (63) to this chemical reaction with the stoichiometric coefficients (125), this gives the following equation for the evolution of the generating function $G\left(z_{A},z_{B};t\right)$:

(126) $$\partial_{t}G\left(z_{A},z_{b};t\right)=\left[z_{B}-z_{A}^{2}\right]\left(K_{1}^{\left(+\right)}\frac{\Omega^{-1}}{2}\frac{\partial^{2}}{\partial z_{A}^{2}}-K_{1}^{\left(-\right)}\frac{\partial }{\partial z_{B}}\right)G\left(z_{A},z_{b};t\right)$$

From Equation (126) one can deduce the equations obeyed by the factorial-moments, the *q*-th factorial moment of a single component is obtained by the following expression, as can be checked easily:

(127) $${\left\{N_{A}^{q}\right\}}_{f}={\left[\frac{\partial^{q}}{\partial z_{A}^{q}}G\left(z_{A},z_{b};t\right)\right]}_{z_{A}=z_{B}=1}=\langle N_{A}\left(N_{A}-1\right)\ldots (N_{A}-q+1)\rangle$$

Also, the bivariate factorial moments ${\left\{N_{A}^{q}N_{B}^{s}\right\}}_{f}$ can be obtained from the generating function $G\left(z_{A},z_{B};t\right)$ by the expression:

(128) $${\left\{N_{A}^{q}N_{B}^{s}\right\}}_{f}={\left[\frac{\partial^{q+s}}{\partial z_{A}^{q}\partial z_{B}^{s}}G\left(z_{A},z_{b};t\right)\right]}_{z_{A}=z_{B}=1}=\langle N_{A}\left(N_{A}-1\right)\ldots \left(N_{A}-q+1\right)N_{B}\left(N_{B}-1\right)\ldots (N_{B}-s+1)\rangle$$

In general application of the operator ${\left[\frac{\partial^{q+s}}{\partial z_{A}^{q}\partial z_{B}^{s}}G\left(z_{A},z_{b};t\right)\right]}_{z_{A}=z_{B}=1}$ to both sides of Equation (126) lead to the evolution equation obeyed by the factorial moment ${\left\{N_{A}^{q}N_{B}^{s}\right\}}_{f}$. It is better to gain efficiency as recommended by Smadbeck and Kadnessis [13], to apply the derivative operator successively and systematically to generate the equations obeyed by all the factorial moments up to a given order. This can be performed with the symbolic operational algebra and calculus tools of MATLAB or MATHEMATICA. For instance, applying the operators $\frac{\partial }{\partial z_{A}}$ to Equation (126) it is obtained the equation:

(129) $$\begin{array}{l} \partial_{t}\frac{\partial }{\partial z_{A}}G\left(z_{A},z_{b};t\right)\\ \;\;\;\;\;\;=\left[-2z_{a}\right]\left(K_{1}^{\left(+\right)}\frac{\Omega^{-1}}{2}\frac{\partial^{2}}{\partial z_{A}^{2}}-K_{1}^{\left(-\right)}\frac{\partial }{\partial z_{B}}\right)G\left(z_{A},z_{b};t\right)\\ \;\;\;\;\;\;+\left[z_{B}-z_{A}^{2}\right]\left(K_{1}^{\left(+\right)}\frac{\Omega^{-1}}{2}\frac{\partial^{3}}{\partial z_{A}^{3}}-K_{1}^{\left(-\right)}\frac{\partial^{2}}{\partial z_{B}\partial z_{A}}\right)G\left(z_{A},z_{b};t\right) \end{array}$$

Setting $z_{A}=z_{B}=1$, in Equation (129) gives the equation for the evolution of the first factorial moment in terms of higher order factorial moments:

(130) $$\partial_{t}{\left\{N_{A}\right\}}_{f}=-K_{1}^{\left(+\right)}\Omega^{-1}{\left\{N_{A}^{2}\right\}}_{f}+2K_{1}^{\left(-\right)}{\left\{N_{B}\right\}}_{f}$$

Applying again the operator $\frac{\partial }{\partial z_{A}}$, to Equation (129) yields the equation:

(131) $$\begin{array}{l} \partial_{t}\frac{\partial^{2}}{\partial z_{A}^{2}}G\left(z_{A},z_{b};t\right)\\ \;\;\;\;\;\;=-2\left(K_{1}^{\left(+\right)}\frac{\Omega^{-1}}{2}\frac{\partial^{2}}{\partial z_{A}^{2}}-K_{1}^{\left(-\right)}\frac{\partial }{\partial z_{B}}\right)G\left(z_{A},z_{b};t\right)\\ \;\;\;\;\;\;-4z_{A}\left(K_{1}^{\left(+\right)}\frac{\Omega^{-1}}{2}\frac{\partial^{3}}{\partial z_{A}^{3}}-K_{1}^{\left(-\right)}\frac{\partial^{2}}{\partial z_{B}\partial z_{A}}\right)G\left(z_{A},z_{b};t\right)\\ \;\;\;\;\;\;+\left[z_{B}-z_{A}^{2}\right]\left(K_{1}^{\left(+\right)}\frac{\Omega^{-1}}{2}\frac{\partial^{4}}{\partial z_{A}^{4}}-K_{1}^{\left(-\right)}\frac{\partial^{3}}{\partial z_{B}\partial z_{A}^{2}}\right)G\left(z_{A},z_{b};t\right) \end{array}$$

Setting again $z_{A}=z_{B}=1$, in Equation (131) yields the evolution equation for the second factorial moment ${\left\{N_{A}^{2}\right\}}_{f}$:

(132) $$\partial_{t}{\left\{N_{A}^{2}\right\}}_{f}=2K_{1}^{\left(-\right)}{\left\{N_{B}\right\}}_{f}+\left(-2K_{1}^{\left(+\right)}\Omega^{-1}{\left\{N_{A}^{2}\right\}}_{f}+4K_{1}^{\left(-\right)}{\left\{N_{A}N_{B}\right\}}_{f}\right)-2K_{1}^{\left(+\right)}\Omega^{-1}{\left\{N_{A}^{3}\right\}}_{f}$$

The idea now is to define an ordered factorial moment column vector ${\left\{N_{1}N_{2}\ldots .N_{n_{s}}\right\}}_{f}$ that contains the lower order moments in the lower index components and the higher order moments in the higher index components. Then, for the case of two components this column vector is defined as the transposed of the following row vector:

(133) $${\left\{N_{A}N_{B}\right\}}_{f}={\left({\left\{N_{A}\right\}}_{f},\;{\left\{N_{B}\right\}}_{f},{\left\{N_{A}^{2}\right\}}_{f},{\left\{N_{A}N_{B}\right\}}_{f},{\left\{N_{B}^{2}\right\}}_{f},{\left\{N_{A}^{3}\right\}}_{f}\;,{\left\{N_{A}^{2}N_{B}\right\}}_{f}\;\;,{\left\{N_{A}N_{B}^{2}\right\}}_{f},{\left\{N_{B}^{3}\right\}}_{f}\;\;,\ldots \right)}^{T},$$

where the upper script *T* denotes the transpose of the row vector to transform it in a column vector. Where in practical application we need to truncate the number of components of this vector at some finite number NC. In general, one obtains an equation for the truncated factorial moment vector. This equation for the evolution of the vector ${\left\{N_{1}N_{2}\ldots .N_{n_{s}}\right\}}_{f}$ was first deduced by Smadbeck and Kaznessis [13], and for the case of the reaction (124), the evolution equation for the column vector of ordered factorial moments, takes de form:

(134) $$\partial_{t}{\left\{N_{A}N_{B}\right\}}_{f}=\left[M_{f}\right]\;{\left\{N_{A}N_{B}\right\}}_{f},$$

where the matrix $\left[M_{f}\right]$, which gives the evolution of the factorial moments for the dimerization reaction and its inverse in a closed system has the components displayed in Table 1. Notice that for the zero order factorial moments, the components are 0. The advantage of using the factorial moment expansion is that the matrix $\left[M_{f}\right]$ is sparser than if one uses an evolution equation for the polynomial moments [13,14]. Also, the bandwidth structure of the matrix for the factorial moments has fewer bands than for the polynomial case. Due to its importance in practical and numerical calculation we discuss in the last section the closure problem.

### 5.3. The Michaelis-Menten Chemical Reaction Network

In this section we study the Michaelis-Menten chemical network, that has been previously studied by several authors [13,57,58] and which is more complex than the previous examples. In this reaction network we have three reactions, in the first one a substrate (S) reacts with an enzyme (E), to form a complex denoted as (E:S). In the second one, this complex degrades back to its original components through the inverse reaction. In the third reaction the complex (E:S) is transformed in a product (P) plus the enzyme (E), so we can write:

(135) $$S+E\begin{array}{c} \overset{K_{1}^{\left(+\right)}}{\to }\\ \underset{K_{1}^{\left(-\right)}}{\leftarrow } \end{array}E:S$$

(136) $$E:S\overset{K_{2}^{\left(+\right)}}{\to }E+P$$

This network has apparently four components and three reactions (reaction 1 plus its reverse reaction and reaction 2). For this network the stoichiometric and the jumps coefficients are:

(137) $$\begin{array}{c} reaction\;1:s_{E}^{\left(1\right)}=1,\;l_{E}^{\left(1\right)}=0,\;\nu_{E}^{\left(1\right)}=-1\\ s_{E:S}^{\left(1\right)}=0,\;l_{E:S}^{\left(1\right)}=1,\;\nu_{E:S}^{\left(1\right)}=+1\\ s_{S}^{\left(1\right)}=1,\;l_{S}^{\left(1\right)}=0,\nu_{S}^{\left(1\right)}=-1 \end{array}$$

(138) $$\begin{array}{c} reaction\;2:\;S_{E:S}^{\left(2\right)}=0,\;l_{E:S}^{\left(2\right)}=0,\;\nu_{E:S}^{\left(2\right)}=-1\\ s_{E}^{\left(2\right)}=0,\;l_{E}^{\left(2\right)}=1,\;\nu_{E}^{\left(2\right)}=+1\\ s_{P}^{\left(2\right)}=0,\;l_{P}^{\left(2\right)}=1,\;\nu_{P}^{\left(2\right)}=+1 \end{array}$$

The next step is to apply Equation (63) to this chemical reaction network with the stoichiometric coefficients (137), and (138), this calculation gives that the generating function $G\left(z_{S},z_{E},\;z_{E:S},\;z_{p};t\right)$, obeys the equation:

(139) $$\begin{array}{l} \partial_{t}G\left(z_{S},z_{E},\;z_{E:S},\;z_{p};t\right)\\ \;\;\;\;\;\;\;\;=\left[z_{E:S}-z_{E}z_{S}\right]\left(\;\;K_{1}^{\left(+\right)}\Omega^{-1}\frac{\partial^{2}}{\partial z_{E}\partial z_{S}}-K_{1}^{\left(-\right)}\frac{\partial }{\partial z_{E:S}}\;\right)G\left(z_{S},z_{E},\;z_{E:S},\;z_{p};t\right)\\ \;\;\;\;\;\;\;\;+\left[z_{E}z_{P}-z_{E:S}\right]K_{2}^{\left(+\right)}\frac{\partial }{\partial z_{E:S}}G\left(z_{S},z_{E},\;z_{E:S},\;z_{p};t\right) \end{array}$$

From Equation (139) it is possible to obtain the equations obeyed by the factorial moments, ${\left\{N_{E}\right\}}_{f},\;{\left\{N_{E}^{2}\right\}}_{f},{\left\{N_{E}N_{S}\right\}}_{f}\ldots .$ applying the operators $\frac{\partial }{\partial z_{E}},\frac{\partial^{2}}{\partial z_{E}^{2}},\;\frac{\partial^{2}}{\partial z_{E}\partial z_{S}},\ldots .$ to Equation (139), and then setting $z_{E}=z_{S}=z_{E:S}=z_{P}=1,$ proceeding in this way, we have obtained the following results for the evolution equations of the factorial moments:

(140) $$\partial_{t}{\left\{N_{E}\right\}}_{f}=\partial_{t}\langle N_{E}\rangle =-K_{1}^{\left(+\right)}\Omega^{-1}\langle N_{E}N_{S}\rangle +K_{1}^{\left(-\right)}\langle N_{E:S}\rangle +K_{2}^{\left(+\right)}\langle N_{E:S}\rangle$$

(141) $$\partial_{t}\;{\left\{N_{E}^{2}\right\}}_{f}=\partial_{t}\langle N_{E}\left(N_{E}-1\right)\rangle =-2K_{1}^{\left(+\right)}\Omega^{-1}\langle N_{E}\left(N_{E}-1\right)N_{S}\rangle +(2K_{1}^{\left(-\right)}+2K_{2}^{\left(+\right)})\langle N_{E:S}N_{E}\rangle$$

(142) $$\begin{array}{l} \partial_{t}{\left\{N_{E}N_{S}\right\}}_{f}=-K_{1}^{\left(+\right)}\Omega^{-1}\langle N_{E}N_{S}\rangle +K_{1}^{\left(-\right)}\langle N_{E:S}\rangle -K_{1}^{\left(+\right)}\Omega^{-1}\langle N_{E}\left(N_{E}-1\right)N_{S}\rangle +\left(2K_{1}^{\left(-\right)}+K_{2}^{\left(+\right)}\right)\langle N_{E:S}N_{S}\rangle \\ \;\;\;\;\;\;\;\;-K_{1}^{\left(+\right)}\Omega^{-1}\langle N_{E}N_{S}\left(N_{S}-1\right)\rangle \end{array}$$

Next, because of the following relations that express the conservation in the total molecule number of substrate and enzyme in their different forms, we can write:

(143) $$N_{S}+N_{E:S}+N_{P}=N_{S_{T}},\;\mathrm{and}\;N_{E}+N_{E:S}=N_{E_{T}}$$

Then, we substitute in Equations (140), (141) and (142), $N_{E:S}$ by $N_{E_{T}}-N_{E}$, and after a little algebra the following results are obtained:

(144) $$\partial_{t}{\left\{N_{E}\right\}}_{f}=\left(K_{1}^{\left(-\right)}+K_{2}^{\left(+\right)}\right)N_{E_{T}}-\left(K_{1}^{\left(-\right)}+K_{2}^{\left(+\right)}\right){\left\{N_{E}\right\}}_{f}-K_{1}^{\left(+\right)}\Omega^{-1}{\left\{N_{E}N_{S}\right\}}_{f}$$

(145) $$\partial_{t}\;{\left\{N_{E}^{2}\right\}}_{f}=\left(2K_{1}^{\left(-\right)}+2K_{2}^{\left(+\right)}\right)\left(N_{E_{T}}-1\right){\left\{N_{E}\right\}}_{f}-\left(2K_{1}^{\left(-\right)}+2K_{2}^{\left(+\right)}\right)\;{\left\{N_{E}^{2}\right\}}_{f}-2K_{1}^{\left(+\right)}\Omega^{-1}\;{\left\{N_{E}^{2}N_{S}\right\}}_{f}$$

(146) $$\begin{array}{ll} \partial_{t}{\left\{N_{E}N_{S}\right\}}_{f} & =K_{1}^{\left(-\right)}N_{E_{T}}-2K_{1}^{\left(-\right)}{\left\{N_{E}\right\}}_{f}+\left(K_{1}^{\left(-\right)}+K_{2}^{\left(+\right)}\right)N_{E_{T}}{\left\{N_{S}\right\}}_{f}-(K_{1}^{\left(+\right)}\Omega^{-1}+K_{1}^{\left(-\right)}+\\ & K_{2}^{\left(+\right)}){\left\{N_{E}N_{S}\right\}}_{f}-K_{1}^{\left(-\right)}\;{\left\{N_{E}^{2}\right\}}_{f}-K_{1}^{\left(+\right)}\Omega^{-1}\left({\left\{N_{E}^{2}N_{S}\right\}}_{f}+{\left\{N_{E}N_{S}^{2}\right\}}_{f}\right) \end{array}$$

where we recall that ${\left\{N_{E}^{2}N_{S}\right\}}_{f}=\langle N_{E}\left(N_{E}-1\right)N_{S}\rangle ,$ and ${\left\{N_{E}N_{S}^{2}\right\}}_{f}=\langle N_{E}N_{S}\left(N_{S}-1\right)\rangle$. Also, we have obtained the evolution equations for the factorial moments $\left\{{N_{S}}_{f}\right\}$ and $\left\{{N_{S}^{2}}_{f}\right\}$ not displayed here for simplicity. Therefore, the matrix $\left[M_{f}\right]$, that gives the evolution of the factorial moments for the Michaelis-Menten reaction network has the components displayed at Table 2.

We must remark that all the terms of the matrix $\left[M_{f}\right]$, which determine the moments evolution for the Michaelis-Menten network are exactly the same that the ones obtained by Smadbeck and Kaznessis [12] except the coefficient of ${\left\{N_{E}\right\}}_{f}$ in the evolution equation for $\partial_{t}{\left\{N_{S}N_{E}\right\}}_{f}$, which is $\left(N_{E_{T}}-2\right)K_{1}^{\left(-\right)}$ in reference [13], and $-2K_{1}^{\left(-\right)}$ in this paper.

### 5.4. The Closure Problem

In this section we discuss the closure problem that consist in approximating the high order moments, which appear in the equations for the moment evolution up to a given order *M*, in terms of lower order moments, this problem has been studied by different author as Ruess et allied [25], Gillespie [15], Grima [16], and many others using different approaches and by by Smalbeck in his PhD thesis [59], using the zero information closure, because this last method is based on the maximum entropy principle will be the first one to be explained. In addition, Ruess et al. have performed a systematic comparison of different closure methods [25], so this paper it is a good starting point for studying this subject.

#### 5.4.1. The Zero-Information Closure

In this subsection we use one component system to simplify the explanation. The closure problem consists in finding a function *F* to express the higher order moments $\mu_{i>M}$ in term of lower ones, so it is possible to write:

(147) $$\mu_{i>M}=F_{i}\left(\mu_{1},\mu_{2},\ldots ,\mu_{M}\right),$$

where if one uses the polynomial moments, which are defined by the expression:

(148) $$\mu_{i}\left(t\right)=\sum_{N=0}^{\infty }N^{i}P\left(N,t\right),$$

and, if we know all the polynomial moments $\mu_{i}\left(t\right)$ of order i≤M at a given time t, then we can build a functional with the information entropy, and the known moments up to order *M* as restrictions as was done in Section 4. In this case we can obtain an approximate PDF that maximizes the information entropy with the M restrictions imposed by the known moments at time t. If we denote this PDF based in the known M polynomial moments with the subscript S, then we may write:

(149) $$P_{S}\left(N,t\right)=\exp(-1+\sum_{k=0}^{M}\lambda_{k}\left(t\right)N^{k})$$

The Lagrange multipliers $\lambda_{k}\left(t\right)$, are obtained solving the following set of equations for the previously known moments $\mu_{i}\left(t\right)$:

(150) $$\mu_{i}\left(t\right)=\sum_{N=0}^{\infty }N^{i}exp(-1+\sum_{k=0}^{M}\lambda_{k}\left(t\right)N^{k}),\;i=0,1,2,\ldots ,M$$

Once we obtain the Lagrange multipliers, we know an approximate expression for the PDF, which is obtained from the first *M*-th moments, and that we denote $P_{S}\left(N,t\right)$ and which is a function of $\mu_{1},\mu_{2},\ldots ,\mu_{M}$. Then, the higher order moments can be expressed in term of lower order ones by means of the expression:

(151) $$\mu_{i>M}^{\left(S\right)}=\sum_{N=0}^{\infty }N^{i}P_{S}\left(N,t\right)=F_{i}\left(\mu_{1},\mu_{2},\ldots ,\mu_{M}\right),$$

The estimation of the higher moments in this way is denoted by the superscript S, that means that the MEP has been used, this closure scheme is known as the zero-information (ZI) closure, and has been recently used by Vlysidis and Kaznessis, using factorial moments for the Slogi chemical reaction network [60]. It does not matter to use one polynomial basis or the factorial moment basis because both polynomial and factorial basis are related by a transformation matrix. 

#### 5.4.2. Different closure methods

In this section we discuss some additional closure methods that have been used in chemical reaction networks. We perform this discussion for a multivariate chemical system with $n_{s}$ different chemical species, as we have seen in previous sections. Fist we define the so called ordinary or normal moments of order *k* for the population of the different chemical species as follows:

(152) $$\mu_{i_{1},i_{2},\ldots ,i_{k}}=\langle N_{i_{1}}N_{i_{2}}\ldots N_{i_{k}}\rangle ,$$

where we have adopted the convention that the indexes are ordered from the smaller ones to the larger ones, $i_{1}\leq i_{2}\leq \ldots \leq i_{k}$, and that any index can be repeated, for instance $\mu_{1,1,2}=\langle N_{1}^{2}N_{2}\rangle$. The second type of moments that will be used are the central moments, these moments are defined as:

(153) $$\mu_{i_{1},i_{2},\ldots ,i_{k}\;}^{\left(c\right)}=\langle (N_{i_{1}}-\langle N_{i_{1}}\rangle )(N_{i_{2}}-\langle N_{i_{2}}\rangle )\ldots (N_{i_{k}}-\langle N_{i_{k}}\rangle )\rangle$$

and the convention index adopted is the same one that for the ordinary moments, which some authors denote as raw moments. The third type of moments introduced are the cumulants [25,40], which are defined as follows:

(154) $$\kappa_{i_{1},i_{2,..,i_{k}}}=\partial_{s_{i1,}s_{i2,\ldots ,}s_{ik}}^{k}G_{\kappa }(s_{1,}s_{2,\ldots ,}s_{n_{s}}{)|}_{s_{1}=s_{2}\ldots .=s_{n_{s}}=0\;,}$$

where $G_{\kappa }\left(s_{1,}s_{2,\ldots ,}s_{n_{s}}\right)$ is the cumulant generating function, which is defined by the following expression [19,40]:

(155) $$G_{\kappa }\left(s_{1,}s_{2,\ldots ,}s_{n_{s}}\right)=log\langle \exp\left(\sum_{i=0}^{n_{s}}s_{i}N_{i}\right)\rangle$$

For instance, to show how Equation (154) works, one can use this expression to obtain the expressions for some low order cumulants in terms of ordinary or normal moments:

(156) $$\kappa_{1}=\partial_{s_{1}}^{1}G_{\kappa }(s_{1,}s_{2,\ldots ,}s_{n_{s}}{)|}_{s_{1}=s_{2}\ldots .=s_{n_{s}}=0}=\langle N_{1}\rangle ,\;\kappa_{2}=\partial_{s_{2}}^{1}G_{\kappa }(s_{1,}s_{2,\ldots ,}s_{n_{s}}{)|}_{s_{1}=s_{2}\ldots .=s_{n_{s}}=0}=\langle N_{2}\rangle ,$$

(157) $$\kappa_{1,1}=\partial_{s_{1},s_{1}}^{2}G_{\kappa }(s_{1,}s_{2,\ldots ,}s_{n_{s}}{)|}_{s_{1}=s_{2}\ldots .=s_{n_{s}}=0}=\langle N_{1}^{2}\rangle -\langle N_{1}\rangle {}^{2},\;\;$$

(158) $$\kappa_{1,2}=\partial_{s_{1},s_{2}}^{2}G_{\kappa }(s_{1,}s_{2,\ldots ,}s_{n_{s}}{)|}_{s_{1}=s_{2}\ldots .=s_{n_{s}}=0}=\langle N_{1}N_{2}\rangle -\langle N_{1}\rangle \langle N_{2}\rangle$$

From the previous equations, it is easily deduced that we can express normal or raw moments in terms of cumulants for instance it is immediately deduced from Equations (156) and (158) that: $\langle N_{1}N_{2}\rangle =\kappa_{1,2}+\kappa_{1}\kappa_{2}$.

With these previous definitions it is easy to stablish several of the most popular closure schemes found in the literature for chemical reaction networks. The first one is the central moment neglect (CMN) moment closure approximation (MCA), which will be denoted by CMN-MCA. If this closure scheme is performed at order *M,* then all the central moments of order larger than *M*, are neglected. Thus, we can write:

(159) $$\mu_{i_{1},i_{2},\ldots .,i_{k}}^{\left(c\right)}=0,\;for\;k>M$$

The second closure scheme, which it is perhaps the most popular one is the “cumulant-neglect MCA”. In this closure scheme if the selected order is *M*, then all cumulant moments of order larger than *M* are neglected. Thus, the following expression can be written:

(160) $$\kappa_{i_{1},i_{2},\ldots ,i_{k}}=0\;for\;k>M$$

For instance, in the cumulant neglect closure of order 2, all cumulants of order larger than two are set to zero, this immediately leads to the result that it is possible to find expressions for the third and higher order normal moments in terms of first and second order ones [19,40], for instance one finds for a two component system that:

(161) $$\mu_{1,2,2}=\mu_{1}\mu_{2,2}+2\mu_{1,2}\mu_{2}-2\mu_{1}\mu_{2}^{2}$$

(162) $$\mu_{1,1,2}=\mu_{2}\mu_{1,1}+2\mu_{1,2}\mu_{1}-2\mu_{2}\mu_{12}^{2}$$

and similar equations are obtained for the rest of third order moments. The next step to closure the problem is to substitute the thirds order moments in the moment evolution equations, by their expression in terms of lower order moments and in this way, we close the hierarchy of moment equations. The “cumulant-neglect MCA” has been used previously in the field of biochemical kinetics by different authors as Gomez-Uribe and Verghese [61], and also by Schnoerr et al. [19].

There are many more closure methods as the Poisson MCA, the log-normal MCA and so on, also there are several software packages as MOCA, StochDynTools, MomentClosure, which contain several methods to close the moments evolution equations [19,61]. 

### 5.5. Study of the Consistency of the Development of the CME in an Order Parameter

In this section we are going to check if the results obtained using the expansion of the CME in an order parameter are consistent with the results obtained using the probability generating function method.

We start with the chemical reaction network formed in an open system by reactions (100) and (101). Instead to expand the Chemical Master Equation in an order parameter, we departure from Equation (112), which gives the evolution equation for the generating function and applying to this equation the operators ${\left[\frac{\partial }{\partial z_{x}}\right]}_{z_{x}=1}$, and ${\left[\frac{\partial^{2}}{\partial z_{x}^{2}}\right]}_{z_{x}=1}$. It is obtained after some calculus that the first and second polynomial moments obey the equations:

(163) $$\frac{d}{dt}\langle N_{x}\rangle =K_{1}^{\left(+\right)}N_{A}+K_{2}^{\left(+\right)}\Omega^{-1}\langle N_{x}\rangle -K_{2}^{+}\Omega^{-1}\langle N_{x}^{2}\rangle$$

(164) $$\frac{d^{2}}{dt^{2}}\langle N_{x}^{2}\rangle =K_{1}^{\left(+\right)}N_{A}+\left[2K_{1}^{\left(+\right)}N_{A}-2K_{2}^{\left(+\right)}\Omega^{-1}\right]\langle N_{x}\rangle +4K_{2}^{\left(+\right)}\Omega^{-1}\langle N_{x}^{2}\rangle -2K_{2}^{\left(+\right)}\Omega^{-1}\langle N_{x}^{3}\rangle$$

The next step is to substitute $N_{x}=\Omega \phi_{x}+\Omega^{1/2}\eta_{x}$, as performed by Van-Kampen in Equations (163) and (164) or $N_{x}=\langle N_{x}\rangle +\Omega^{1/2}{\bar{\eta }}_{x}$ as performed by Andreychenko et al. [30].

Performing first the substitution $N_{x}=\Omega \phi_{x}+\Omega^{1/2}\eta_{x}$ in (163) and collecting all the terms of order $\Omega^{0}$, yields the following macroscopic kinetic equation:

(165) $$\frac{d}{dt}\phi_{x}=K_{1}^{\left(+\right)}\phi_{A}-K_{2}^{\left(+\right)}\phi_{x}^{2}$$

Collecting now the terms of order $\Omega^{1/2}$, gives the result:

(166) $$\partial_{t}\langle \eta_{x}\rangle =-2K_{2}^{\left(+\right)}\langle \eta_{x}\rangle$$

Equations (165) and (166) are the same ones that the ones obtained from Van Kampen expansion of the Master Equation. If we perform now the substitution $N_{x}=\Omega \phi_{x}+\Omega^{1/2}\eta_{x}$, in (164) and we use the following identities:

(167) $$\langle N_{x}^{2}\rangle ={\langle (\Omega \phi_{x}+\Omega^{1/2}\eta_{x})}^{2}\rangle ={\left(\Omega \phi_{x}\right)}^{2}+2\Omega^{3/2}\phi_{x}\langle \eta_{x}\rangle +\Omega \langle \eta_{x}^{2}\rangle$$

(168) $$\langle N_{x}^{3}\rangle ={\langle (\Omega \phi_{x}+\Omega^{1/2}\eta_{x})}^{3}\rangle ={\left(\Omega \phi_{x}\right)}^{3}+3\;\Omega^{5/2}{\left(\phi_{x}\right)}^{2}\langle \eta_{x}\rangle +3\;\Omega^{2}\phi_{x}\langle \eta_{x}^{2}\rangle +\Omega^{3/2}\langle \eta_{x}^{3}\rangle$$

Because of Equations (167) and (168), the following result is obtained from Equation (164), collecting the terms of order Ω:

(169) $$\partial_{t}\langle \eta_{x}^{2}\rangle =-6K_{2\;}^{+}\phi_{x}\left(t\right)\langle \eta_{x}^{2}\rangle +\left(K_{1}^{\left(+\right)}\phi_{A}+4K_{2}^{\left(+\right)}\phi_{x}^{2}\right)$$

The terms in this case are the same ones that in Van Kampen approach but now some of the coefficients are different as it is deduced comparing Equations (169) and (111).

Let us use now, the alternative recently proposed by Andrechenko et al. [49], in this case they propose to use $N_{x}=\Omega {\bar{\phi }}_{x}+\Omega^{1/2}{\bar{\eta }}_{x}$ with ${\bar{\phi }}_{x}=\frac{\langle N_{x}\rangle }{\Omega }$. The great advantage of this formulation is that $\langle {\bar{\eta }}_{x}\rangle =0$, as was mentioned previously in Section 5.1. Because of this property we can write:

(170) $$\langle N_{x}^{2}\rangle =\langle N_{x}\rangle {}^{2}+\Omega \langle {\bar{\eta }}_{x}{}^{2}\rangle ={\left(\Omega {\bar{\phi }}_{x}\right)}^{2}+\Omega \langle {\bar{\eta }}_{x}{}^{2}\rangle$$

(171) $$\langle N_{x}^{3}\rangle ={\left(\Omega {\bar{\phi }}_{x}\right)}^{3}+3\Omega^{2}{\bar{\phi }}_{x}\;\langle {\bar{\eta }}_{x}{}^{2}\rangle +\Omega^{3/2}\langle {\bar{\eta }}_{x}{}^{3}\rangle$$

Performing first the substitution $N_{x}=\Omega {\bar{\phi }}_{x}+\Omega^{1/2}{\bar{\eta }}_{x}$ in (163) and collecting all the terms of order $\Omega^{0}$, yields the following macroscopic kinetic equation:

(172) $$\frac{d}{dt}{\bar{\phi }}_{x}=K_{1}^{\left(+\right)}\phi_{A}-K_{2}^{\left(+\right)}{\bar{\phi }}_{x}{}^{2}$$

Collecting the terms of order $\Omega^{1/2}$ gives:

(173) $$\partial_{t}\langle {\bar{\eta }}_{x}\rangle =0$$

In this case, because of (170) and (171), the following result, is obtained from equation (164) collecting the terms of order Ω:

(174) $$\partial_{t}\langle {\bar{\eta }}_{x}{}^{2}\rangle =-4{\bar{\phi }}_{x}\left(t\right)\langle {\bar{\eta }}_{x}{}^{2}\rangle +\left(K_{1}^{\left(+\right)}\phi_{A}+2K_{2}^{\left(+\right)}{\bar{\phi }}_{x}{}^{2}\right)$$

We observe that the macroscopic Equations (165) and (172) are the same ones that the ones obtained by Van-Kampen method of expansion of the master equation. Also, Equation (166) for the first moment of the fluctuations when we use $N_{x}=\Omega \phi_{x}+\Omega^{1/2}\eta_{x}$, is also the same. But for the second moment evolution equation of the fluctuations, Equation (174), the terms are the same in Andreychenko approach $N_{x}=\langle N_{x}\rangle +\Omega^{1/2}{\bar{\eta }}_{x}$, but some coefficients are different if $N_{x}=\Omega \phi_{x}+\Omega^{1/2}\eta_{x}$, as it is observed in Equation (169) for the second moment of the fluctuations.

## 6. Discussion and Conclusions

The application of methods based on the theory of stochastic processes has become very popular in chemistry and biological systems, especially since the development of the Chemical Master Equation (CME), and the Monte-Carlo methods to solve properly the CME by sampling over the probability distributions [4,6,7,8]. In addition, the introduction of the finite state projection method (FSP) by Munsky and Khammash, which provides a direct solution of the CME at a desired degree of approximation without generating Monte-Carlo simulations, has supplied an alternative way to Monte-Carlo methods [12]. Since the pioneering works of Van Kampen [8] and Nicolis and Prigogine [4], the developed methods and the range of applications of the CME have experienced a noteworthy increase. This is especially true for the methods based on Monte-Carlo as the Sampling Stochastic Approach (SSA) of Gillespie, which performs a sampling over the underlying probability distributions. It is necessary to mention the methods based on the Chemical Fokker-Planck (CFP) equation, which can be obtained performing a Kramers-Moyal expansion in the Chemical Master equation and truncating this expansion to second order, in this way one obtains the CFP Equation. However as pointed out by Gardiner (p. 267, [40]) this is only an approximation whose asymptotic expansion is identical to order $\Omega^{-1}$ with that of the CME.

More recently people as Smadbeck, Kaznessis, Sotiropoulos, Vlysidis and others [13,14,59] have tried to solve the moment equations and to reconstruct the PDF from the moments applying the maximum entropy principle. The problem of this approach is that the moment order must be truncated at some value, so one needs a method to compute and truncate in a consistent way the hierarchy of moments. Because if not the number of differential equations to solve becomes too large, and many moments are necessary to obtain a good approximation to the probability distribution. There have been many attempts in the past to find a consistent methodology to truncate the moment hierarchy; some of these methods as the “cumulant-neglect MCA” are reviewed in this paper [20,40,62]. At this point the zero-information truncation or ZI truncation provides a methodology to obtain in a consistent way the truncated moments in terms of lower ones; this method gives similar results than the SSA method with the improvements to this method performed by Gibson and Bruck [63].

In this review we have tried to present a unified vision of the chemical reaction networks and the existing methods to compute the probability distribution of the different chemical compounds that enter in the network. We have started by the root basis of chemical kinetics from a microscopic point of view to compute properly the reaction rates of the different chemical reactions that are necessary to obtain the chemical propensities that enter the chemical master equation as a fundamental cornerstone. We have centered the analysis in the bimolecular reactions computing from a microscopic basis the reaction rates, that are necessary to obtain the chemical propensities. 

The next step has been to obtain the chemical master equation (CME), traditionally this equation is deduced from the Chapman-Kolmogorov equation, and we have used this way to obtain the master equation. In addition, we have considered very illustrative to deduce the chemical master equation from a probability balance that capture better the physical roots of the problem. Then we have discussed the limitations of the CME, first we have reviewed the two fundamental conditions, which must hold to be valid the application of the CME in diffusion-limited systems, especially when bimolecular reactions occurs. These conditions were studied by Gillespie et al. [41], and are that the reactant molecules in the reacting system are at the same time “dilute” and “well-mixed”. In addition, it is important to know the factors which influence the spatial correlations to understand the limitations of the CME; this influence is studied through the two point correlation function in Section 2.5, it is found that correlations can be important when the chemical system is far from equilibrium and in addition the correlation length is large.

Then, we present two alternative methods that are used to obtain the probability distribution of the chemical species of the reaction network, one is the expansion of the chemical master equation in an order parameter that is the system volume and the other method is the probability generating function method. To see if both methods are equivalent we have performed a consistency analysis of both methods in Section 5.5, and we have found that although the results of both methods for the macroscopic evolution equations and for the first moment of the fluctuations are similar, some differences can exists in the coefficients of the second moment equation depending on the way that is performed the splitting of the random variables.

Then, we have deduced the expansion of the chemical master equation in the order parameter using a multinomial expansion of the jump operators, $E_{j}^{\nu_{j}^{\left(r\right)}}$. The equation obtained gives the same results than Van-Kampen expansion method of the Master Equation for some simple reaction network studied in Section 5.1 and Section 5.2.

Because we can compute the moments of the distribution of the chemical species then it is important to reconstruct the PDF from these moments. The method to obtain the unknown PDF from the moments is based on the application of the maximum information entropy principle (MEP). The main drawback of this methods is that requires to solve a set of non-linear equations to obtain the values of the Lagrange multipliers that are present in the exponent of the PDF expression. Three types of basis can be used to reconstruct the PDF, the polynomial basis, the factorial moment basis and the cumulant basis. The factorial moments evolution equations can be obtained directly from the generating function evolution equation as was shown in Section 5.1, Section 5.2 and Section 5.3. Performing some algebra is possible to obtain the polynomial moments from the factorial moments. The polynomial moments and the factorial moments as shown by Smadbeck and Kaznessis [13] are both related by a similarity transform matrix. Other important basis, is the cumulant basis, the interest of this basis is that the cumulants have cluster properties, in the sense that for independent random variables all the cumulant vanish, notice that the factorial moments do not enjoy this property [40]. Also, it is possible to express the polynomial moments in terms of cumulants. So, we can pass from one basis to the other. 

The expansion of the master equation in an order parameter and the multiplicity generating function method are applied to several simple univariate and multivariate chemical reaction networks. First the multinomial expansion formula derived in Section 3, is applied to a non-linear simple reaction network and the resulting equation for the evolution of the macroscopic variable and the moments of the fluctuation are compared with Van-Kampen results [8]. It is found that the results are the same. Then this same case is studied by the probability generating function method (PGFM), by application of this method, see also Section 5.1, we obtained the equations obeyed by the factorial moments, at this point a study of the self-consistency of the PGFM is performed in the equations obeyed by the factorial moments when the system is Poissonian and the method is self-consistent as explained at the end of Section 5.1. The next step was to check if the two previous methods, the PGFM and the expansion in an order parameter of the CME, were cross-consistent i.e., give the same results. This study was performed in Section 5.5. Instead to perform a systematic expansion in an order parameter we obtain first the equations for the evolution of the factorial moments, of first order, second order and so on. And at posteriori, we perform the substitution $N_{x}=\Omega \phi_{x}+\Omega^{1/2}\eta_{x}$, as performed by Van-Kampen in Equations (163) and (164) or $N_{x}=\Omega N_{x}+\Omega^{1/2}{\bar{\eta }}_{x}$ as performed by Andreychenko et al [49]. Then we collect the terms of the same order in the expansion, and the result yields the correct macroscopic equation, the same equation for the evolution of the first moment of the fluctuations, but for the second moment $\langle \eta_{x}^{2}\rangle$, the equation have the same terms than in Van-Kampen results for this same case [8], but two of the terms has a different coefficient in the approach $N_{x}=\Omega \phi_{x}+\Omega^{1/2}\eta_{x}$. However, the equation for the second moment was the same in Andreychenko et al. approach [49]. This cross checking shows that both methods are not completely equivalents, and that further research is necessary.

Then in Section 5.2, we apply the PGFM to a two-component chemical reaction network studied by Smadbeck, and Kaznessis [12], that consisted in the reversible dimerization of two monomers. Also, in Section 5.3 we have applied the PGFM method to the Michaelis-Menten reaction network. We used the factorial moment basis for both cases, and the results were the same ones that the ones obtained by these authors, except one small difference in one term for the Michaelis-Menten network. Finally, in Section 5.4 we have reviewed the closure problem studying some classical closure methods as the “cumulant neglect MCA” and the recent advances in the ZI closure method, as a systematic and consistent approach to perform the closure of the moment hierarchy at the desired truncation order.

## Figures and Tables

**Table 1:** Components of the matrix $\left[M_{f}\right]$, which determines the factorial moments evolution of the dimerization reaction network.

	${\left\{N_{A}\right\}}_{f}$	${\left\{N_{B}\right\}}_{f}$	${\left\{N_{A}^{2}\right\}}_{f}$	${\left\{N_{A}N_{B}\right\}}_{f}$	${\left\{N_{B}^{2}\right\}}_{f}$	${\left\{N_{A}^{3}\right\}}_{f}$	${\left\{N_{A}^{2}N_{B}\right\}}_{f}$
$\partial_{t}{\left\{1\right\}}_{f}$	0	0	0	0	0	0	0
$\partial_{t}{\left\{N_{A}\right\}}_{f}$	0	$2K_{1}^{\left(-\right)}$	$-K_{1}^{\left(+\right)}\Omega^{-1}$	0	0	0	0
$\partial_{t}{\left\{N_{B}\right\}}_{f}$	0	$-K_{1}^{\left(-\right)}$	$0.5\;K_{1}^{\left(+\right)}\Omega^{-1}$	0	0	0	0
$\partial_{t}{\left\{N_{A}^{2}\right\}}_{f}$	0	$2K_{1}^{\left(-\right)}$	$-K_{1}^{\left(+\right)}\Omega^{-1}$	$4K_{1}^{\left(-\right)}$	0	$-2K_{1}^{\left(+\right)}\Omega^{-1}$	0
$\partial_{t}{\left\{N_{A}N_{B}\right\}}_{f}$	0	0	0	$-K_{1}^{\left(-\right)}$	$2K_{1}^{\left(-\right)}$	$0.5\;K_{1}^{\left(+\right)}\Omega^{-1}$	$-K_{1}^{\left(+\right)}\Omega^{-1}$
$\partial_{t}{\left\{N_{B}^{2}\right\}}_{f}$	0	0	0	0	$-2K_{1}^{\left(-\right)}$	0	$K_{1}^{\left(+\right)}\Omega^{-1}$

**Table 2:** Components of the matrix $\left[M_{f}\right]$, which determines the factorial moments evolution of the Michaelis-Menten reaction network.

	${\left\{1\right\}}_{f}$	${\left\{N_{S}\right\}}_{f}$	${\left\{N_{E}\right\}}_{f}$	${\left\{N_{S}^{2}\right\}}_{f}$	${\left\{N_{S}N_{E}\right\}}_{f}$	${\left\{N_{E}^{2}\right\}}_{f}$	${\left\{N_{S}^{3}\right\}}_{f}$	${\left\{N_{S}^{2}N_{E}\right\}}_{f}$	${\left\{N_{E}^{2}N_{S}\right\}}_{f}$
$\partial_{t}{\left\{1\right\}}_{f}$	0	0	0	0	0	0	0	0	0
$\partial_{t}{\left\{N_{S}\right\}}_{f}$	$K_{1}^{\left(-\right)}N_{E_{T}}$	0	$-K_{1}^{\left(-\right)}$	0	$-K_{1}^{\left(+\right)}\Omega^{-1}$	0	0	0	0
$\partial_{t}{\left\{N_{E}\right\}}_{f}$	$\left(K_{1}^{\left(-\right)}+K_{2}^{\left(+\right)}\right)\;N_{E_{T}}$	0	$-K_{1}^{\left(-\right)}-K_{2}^{\left(+\right)}$	0	$-K_{1}^{\left(+\right)}\Omega^{-1}$	0	0	0	0
$\partial_{t}{\left\{N_{S}^{2}\right\}}_{f}$	0	$2K_{1}^{\left(-\right)}N_{E_{T}}$	0	0	$-2K_{1}^{\left(-\right)}$	0	0	$-2K_{1}^{\left(+\right)}\Omega^{-1}$	0
$\partial_{t}{\left\{N_{S}N_{E}\right\}}_{f}$	$K_{1}^{\left(-\right)}N_{E_{T}}$	$(K_{1}^{\left(-\right)}+K_{2}^{\left(+\right)})\;N_{E_{T}}$	$-2K_{1}^{\left(-\right)}$	0	$K_{1}^{\left(+\right)}\Omega^{-1}+K_{1}^{\left(-\right)}+K_{2}^{\left(+\right)}$	$-K_{1}^{\left(-\right)}$	0	$-K_{1}^{\left(+\right)}\Omega^{-1}$	$-K_{1}^{\left(+\right)}\Omega^{-1}$
$\partial_{t}{\left\{N_{E}^{2}\right\}}_{f}$	0	0	$2\left(N_{E_{T}}-1\right)\left(K_{1}^{\left(-\right)}+K_{2}^{\left(+\right)}\right)$	0	0	$-2\left(K_{1}^{\left(-\right)}+K_{2}^{\left(+\right)}\right)$	0	0	$-2K_{1}^{\left(+\right)}\Omega^{-1}$
